# Secretome signature of cardiopoietic cells echoed in rescued infarcted heart proteome

**DOI:** 10.1002/sctm.20-0509

**Published:** 2021-05-28

**Authors:** D. Kent Arrell, Ruben J. Crespo‐Diaz, Satsuki Yamada, Ryounghoon Jeon, Armin Garmany, Sungjo Park, Jeffrey P. Adolf, Christopher Livia, Matthew L. Hillestad, Jozef Bartunek, Atta Behfar, Andre Terzic

**Affiliations:** ^1^ Center for Regenerative Medicine, Marriott Heart Disease Research Program Van Cleve Cardiac Regenerative Medicine Program, Mayo Clinic Rochester Minnesota USA; ^2^ Department of Cardiovascular Medicine Mayo Clinic Rochester Minnesota USA; ^3^ Department of Molecular Pharmacology & Experimental Therapeutics Mayo Clinic Rochester Minnesota USA; ^4^ Cardiovascular Division University of Minnesota Minneapolis Minnesota USA; ^5^ Division of Geriatric & Gerontology Medicine Mayo Clinic Rochester Minnesota USA; ^6^ Mayo Clinic Alix School of Medicine, Mayo Clinic Graduate School of Biomedical Sciences Rochester Minnesota USA; ^7^ Cardiovascular Center, OLV Hospital Aalst Belgium; ^8^ Department of Physiology & Biomedical Engineering Mayo Clinic Rochester Minnesota USA; ^9^ Department of Clinical Genomics Mayo Clinic Rochester Minnesota USA

**Keywords:** cardiopoiesis, clinomics, heart failure, regenerative medicine, stem cells, systems biology, therapy

## Abstract

Stem cell paracrine activity is implicated in cardiac repair. Linkage between secretome functionality and therapeutic outcome was here interrogated by systems analytics of biobanked human cardiopoietic cells, a regenerative biologic in advanced clinical trials. Protein chip array identified 155 proteins differentially secreted by cardiopoietic cells with clinical benefit, expanded into a 520 node network, collectively revealing inherent vasculogenic properties along with cardiac and smooth muscle differentiation and development. Next generation RNA sequencing, refined by pathway analysis, pinpointed miR‐146 dependent regulation upstream of the decoded secretome. Intracellular and extracellular integration unmasked commonality across cardio‐vasculogenic processes. Mirroring the secretome pattern, infarcted hearts benefiting from cardiopoietic cell therapy restored the disease proteome engaging cardiovascular system functions. The cardiopoietic cell secretome thus confers a therapeutic molecular imprint on recipient hearts, with response informed by predictive systems profiling.

## Graphical abstract

Reverse translational decoding characterized the secretome of cardiopoietic cells, a regenerative therapeutic in clinical testing. A cardiovasculogenic systems signature distinguished high from low response profiles, an imprint echoed in the restored diseased proteome. Linkage of realized outcome with secretome identity suggests paracrine centrality in regenerative fitness. Pre‐intervention secretome profiling would thus inform optimized selection of regenerative biologic candidates.

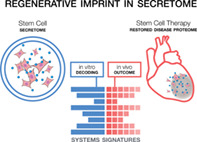

**Significance statement**

The secretome of clinical trial‐biobanked cardiopoietic cells was here decoded. The mined (cardiomyo)vasculogenic systems signature was echoed in the response of cell recipients demonstrating disease rescue. The present clinomics study links innate secretome traits with outcome.

**Significance statement**

The secretome of clinical trial‐biobanked cardiopoietic cells was here decoded. The mined (cardiomyo)vasculogenic systems signature was echoed in the response of cell recipients demonstrating disease rescue. The present clinomics study links innate secretome traits with outcome.

## 1 INTRODUCTION

Cardiopoietic cells, developed for ischemic heart failure treatment, have reached clinical testing, showing promise in select patient populations.^1^, ^2^, ^3^, ^4^ While mode of action remains uncertain for stem cell‐based therapies, limited integration of delivered cells into infarcted hearts suggests paracrine contribution.^5^, ^6^ The association of therapeutic outcome with cardiopoietic cell secretome identity remains, however, unexplored.

Mixed results observed in stem cell clinical trials provide an opportunity to probe for determinants of outcome.^7^, ^8^ Here, leveraging high vs low response cohorts, a systems interrogation of the composition and functionality of the differential cardiopoietic cell secretome were surveyed, in tandem with upstream intracellular regulators induced by the cardiopoiesis process. Reverse translational decoding offered insight into the paracrine imprint underlying therapeutic benefit.

## 2 MATERIALS AND METHODS

### 2.1 Cells

Under regulatory and ethics approval, cardiopoietic cells were generated from recombinant growth factor cocktail‐primed bone marrow mesenchymal stromal cells.^9^ Derived progeny were immunoprobed for cardiac transcription factor expression to authenticate cellular phenotype purity.^10^ Cell donors were patients with documented ischemic heart failure, receiving optimal standard‐of‐care therapy and undergoing clinical trial evaluation, including demographics and comorbidity profiling with ejection fraction on echocardiography used as an efficacy readout.^9^ Aliquots from ≥5 distinct cell lines, fulfilling predetermined trial quality release criteria, were biobanked and processed to isolate total RNA for microRNA (miRNA) profiling or cultured to yield conditioned media collected for protein array scanning of secretome. Primary analysis and validation were conducted in independent and investigator‐blinded fashion.

### 2.2 Molecular profiling

Following cardiopoietic induction,^9^ cells at ≈80% confluency were washed and incubated 48 hours without serum. Cell viability was assessed by morphology and Trypan blue exclusion. Centrifugation‐derived (1000*g*, 10 minutes) conditioned media supernatant was dialyzed against PBS (1:2500) prior to secretome analysis by protein chip array (RayBio Human Antibody Array L507; RayBiotech), quantified on a GenePix 4000B scanner with output normalized to corresponding cell total protein content. Small RNA libraries were prepared from total RNA (NEBNext Multiplex Small RNA Kit, New England Biolabs), reverse transcribed into a cDNA library, amplified, and assessed for miRNA by next generation sequencing. Libraries were sequenced (Illumina HiSeq 2000, TruSeq SBS sequencing kit), base‐calling performed (Illumina RTA v.1.12.4.2), aligned to the reference genome hg19 and miRBase (Bowtie), and quantified (miRDeep2). With low read miRNAs filtered, differential expression was conducted (edgeR) using Benjamini‐Hochberg false discovery rate (B‐H FDR) correction and agglomeratively clustered (ClustVis, https://biit.cs.ut.ee/clustvis/).

### 2.3 Pathway and network analysis

Differential secretome was interrogated by Ingenuity Pathway Analysis (IPA) for functional sub‐annotations, upstream regulators, and network generation. Significance was calculated using Fisher's exact test with B‐H FDR correction, and z‐score transformed as appropriate. Collective evaluation was carried out at network level using IPA for functional annotation, with network interactions exported to Cytoscape (v.3.8.2) for node topology parameter assessment using NetworkAnalyzer^11^ and Bioinformatic Network Gene Ontology (BiNGO) to interpret ontological enrichment of biological processes.^12^ Pairwise interactions were visualized as an undirected adjacency matrix using the Python package Seaborn.

### 2.4 Infarcted heart assessment

With Institutional Animal Care and Use Committee approval, nude mice were infarcted and at 1‐month postinfarction randomized into untreated or cardiopoietic cell treated cohorts. One‐month post‐randomization, multi‐parametric outcomes measured in blinded fashion included: left ventricular structure and ejection fraction (EF) by 2D B‐mode echocardiography^13^; degree and extent of akinesis using myocardial deformation imaging^14^ (Vevo Strain and Python); plasma N‐terminal pro‐atrial natriuretic peptide (NT‐proANP, BI‐2089, BioMedica) level; and CD31 (AF3628, R&D) plus 4′,6‐diamidino‐2‐phenylindole (DAPI, H‐1200‐10, VECTASHIELD) staining.^15^ Significance (*P* < .05) was evaluated by nonparametric Mann‐Whitney *U* test and repeated measures ANOVA. Proteome was extracted from disease severity titrated infarcted hearts,^16^ and analyzed by label‐free peptide quantification following nano‐flow liquid chromatography electrospray tandem mass spectrometry.^17^ Protein identities were assigned and quantified using MaxQuant v.1.5.1.2, and differential expression calculated by two‐sided ANOVA with Gaussian linked function using R.^17^ Cohort level differences were evaluated in IPA, using Fisher's exact test with B‐H FDR correction to identify enriched cardiac adverse effects and prioritized cardiovascular system development functions.

## 3 RESULTS

### 3.1 Cardiopoietic cell secretome

**FIGURE 1:**
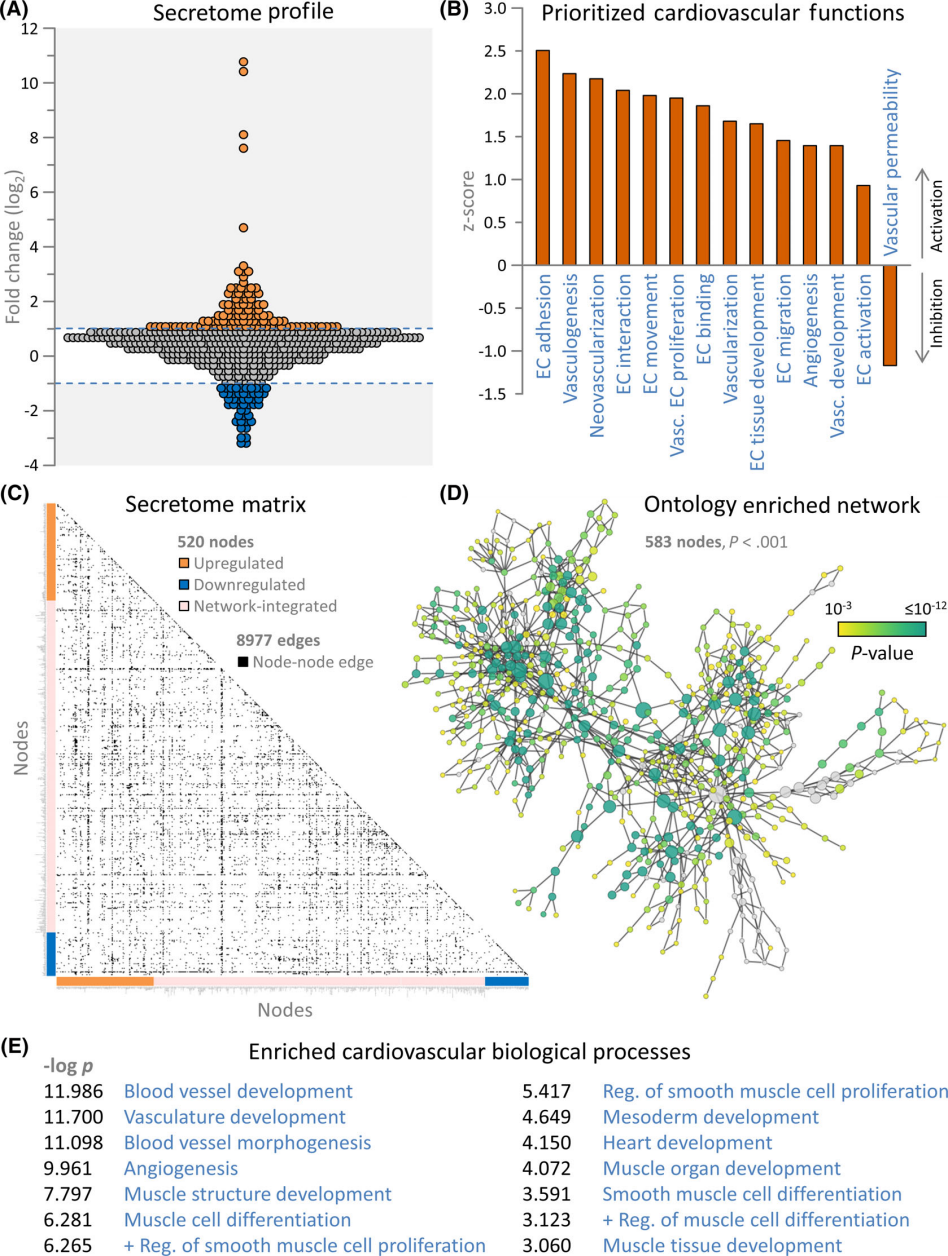
Cardiopoietic secretome harbors pro‐cardiovasculogenic traits. A, Conditioned media was assessed by biotin labeling on a 507 protein streptavidin‐conjugated fluorescent chip array, indicating that effective cardiopoietic cells secreted 155 differentially expressed proteins at >2‐fold change (107 upregulated—orange; 48 downregulated—blue). B, Ingenuity Pathway Analysis (IPA) for enriched cardiovascular developmental functions revealed vascular development, angiogenesis, vasculogenesis, and multiple endothelial cell (EC) functions as prioritized annotations associated with the differential secretome, with positive z‐scores representing increased likelihood of activation and negative scores increased likelihood of inhibition. C, IPA integration of the differential secretome generated an interaction network comprising 520 nodes, clustered along matrix axes by inclusion basis (colored bars), with 8977 pairwise connections (edges) denoted as black squares. Node names and topological properties are listed in supporting Table S1. The top 10 IPA cardiovascular system development functional annotations associated with the network were angiogenesis, vascular development, vasculogenesis, EC interaction, EC binding, endothelial tissue development, cardiovascular tissue development, vascular EC interaction, EC development, and EC adhesion (all *P* < 1 × 10^−14^). D, In parallel, network assessment by Biological Network Gene Ontology (BiNGO) to prioritize enriched biological processes, using hypergeometric distribution with Benjamini‐Hochberg false discovery rate correction, revealed a 660 node hierarchical ontology network, with 583 significantly enriched (*P* < .001), and the top 100 presented in supporting Table S2. E, Enriched cardiovascular biological processes within the BiNGO analysis relate to vascular, angiogenic, and smooth and cardiac muscle development (Positive regulation = + Reg.)

Profiled by chip array, conditioned media revealed 155 proteins differentially released (>2‐fold up or down; Figure 1A) from clinical trial biobanked cardiopoietic cells with high vs low therapeutic response.^9^ Differential secretome pathway analysis prioritized, within cardiovascular system development and functions, activation of vasculogenic, angiogenic, and endothelial cell development (Figure 1B). Cohesiveness of vascular functionality was further demonstrated within the integrated secretome network (Figure 1C; supporting Table S1). Neovasculogenesis and smooth/cardiac muscle development were also prioritized from cardiovascular biological process enrichment (Figure 1D,E; supporting Table S2). Thus, cardiopoietic cells with higher efficacy exhibit a distinguishing secretome signature, with cardio‐vasculogenic functionality predicted at the protein and expanded network levels.

### 3.2 Integrated miRNome confers secretome functionality

**FIGURE 2:**
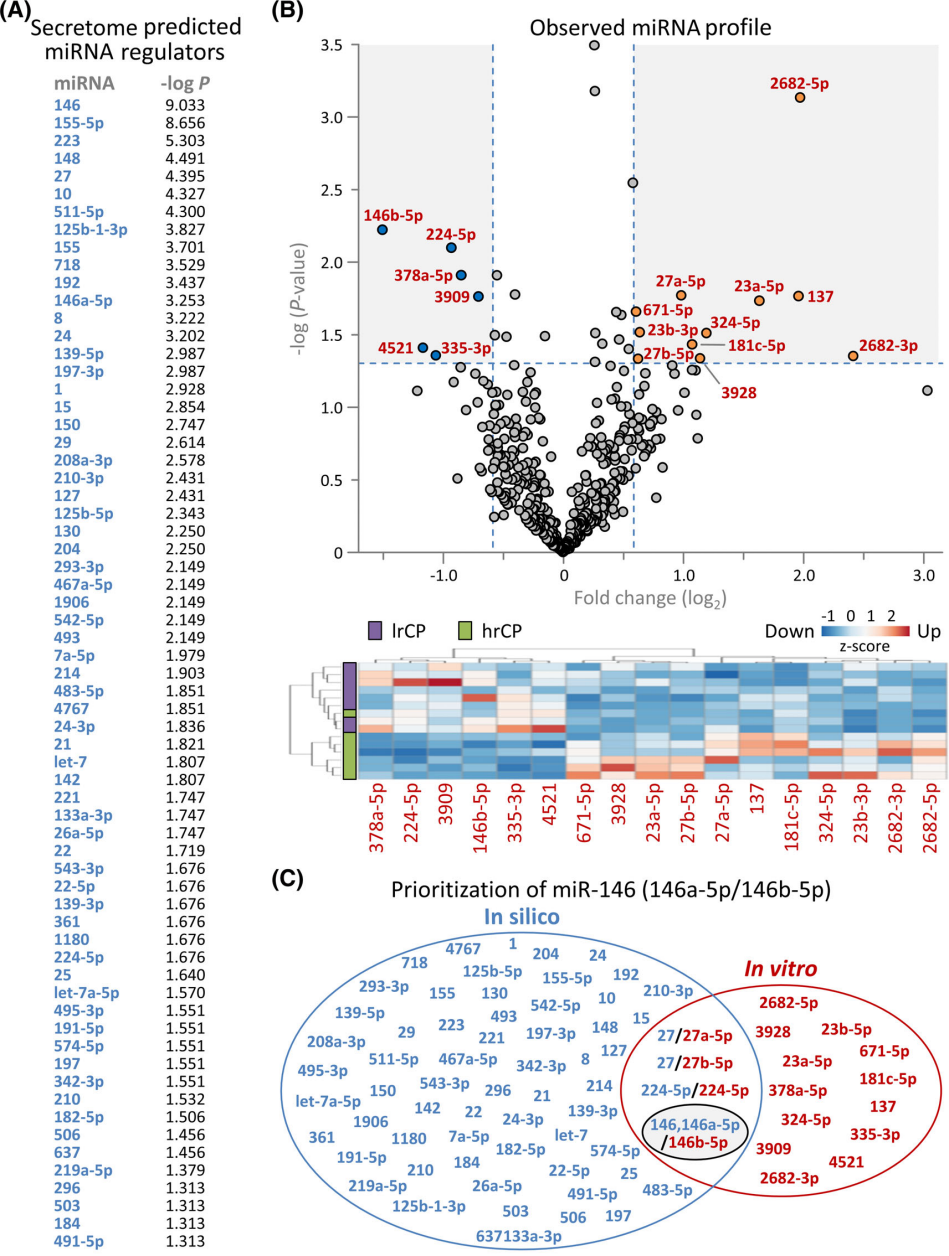
Cardiopoietic secretome miRNA regulators. A, Differential secretome interrogation by Ingenuity Pathway Analysis identified 65 miRNAs as upstream candidate regulators induced by the cardiopoietic process, with individual identities rank ordered by corresponding enrichment *P*‐value. B, Independently, the miRNA makeup of high vs low response cardiopoietic cells was evaluated by miRNA‐Seq detecting 447 discrete miRNAs, of which 17 were differentially expressed at >1.5‐fold change, *P* < .05, as visualized by volcano plot (B, upper, with 11 upregulated—orange; 6 downregulated—blue). Differential miRNA clustering segregated high vs low response cardiopoietic cells (hrCP and lrCP, respectively), as visualized by agglomerative hierarchical heatmap (B, lower, with differentially expressed miRNAs listed). For each miRNA, z‐scores represent intensity for individual samples normalized to mean intensity. C, Overlap between the 65 candidate miRNA regulators of the secretome (blue) and the 17 differentially expressed miRNAs of hrCP (red) revealed a subset of 4 shared miRNAs, visualized by Venn diagram. miR‐146, 146a‐5p/146b‐5p (black ellipse) exhibited consistent directionality, with observed downregulation in miRNA‐Seq and predicted inhibition based on the secretome profile. In fact, it presented as the most extensively enriched (miR‐146, *P* = 9.27 × 10^−10^) of predicted upstream miRNA regulators and had the greatest extent of observed downregulated change (miR‐146b‐5p, 2.84‐fold, *P* = 5.97 × 10^−3^) among cohorts

**FIGURE 3:**
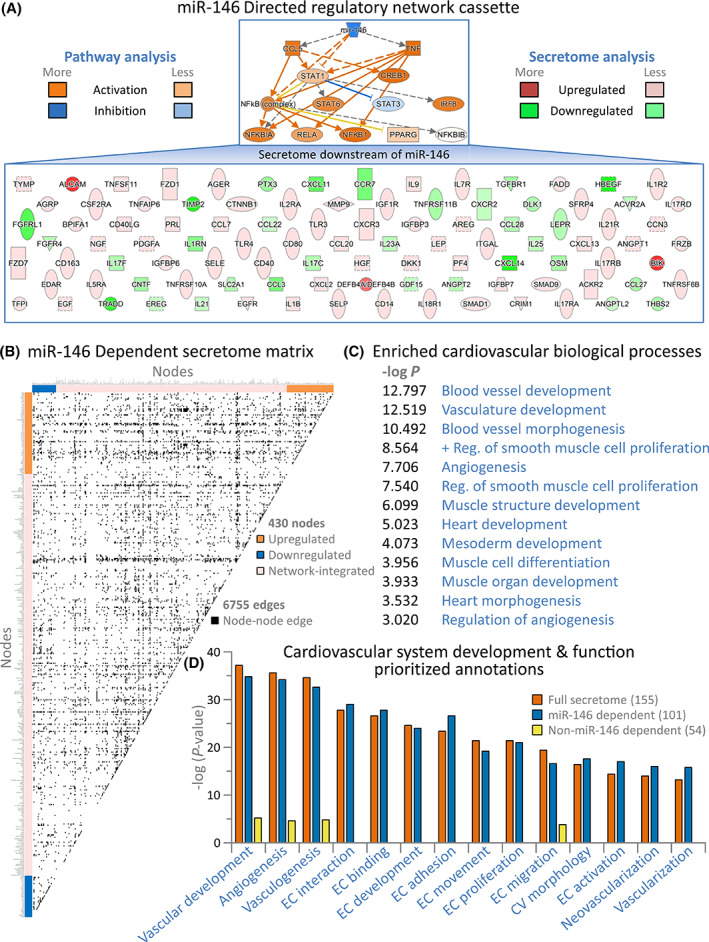
Secretome functionality concentrated within the miR‐146 dependent sub‐secretome. (A) Pathway interrogation pinpointed miR‐146 at the apex of a 14‐protein network upstream of the secretome (A, upper) comprising interdependent transcriptional regulators (TNFα; CREB1; PPARγ; CCL5; IRF8; STAT1; STAT3; STAT6; and the NFκB complex, including NFκB1, NFκBIA, NFκBIB, and RELA) predicted to be activated or inhibited (orange or blue, respectively). Each node of the miR‐146 directed network regulates one or more downstream components of a cardiopoietic sub‐secretome (A, lower), comprising 65% of the overall differential secretome (101 of 155 proteins), with protein increase or decrease shown (red or green, respectively). B, Integrating the 101 protein miR‐146 dependent sub‐secretome into an expanded interaction neighborhood generated a 430 node network, clustered along matrix axes by inclusion basis (colored bars), connected by 6755 interactions (edges) shown as black squares. Network node names and topological parameters are listed in supporting Table S3. C, BiNGO analysis of the miR‐146 dependent secretome network yielded 558 significantly enriched terms (*P* < .001; with the top 100 listed in supporting Table S4), and revealed enriched cardiovascular biological processes related to vascular, angiogenic, and smooth/cardiac muscle development (Positive regulation = + Reg.). D, Enriched cardiovascular system development and functions of the full secretome (orange) and the miR‐146 dependent (blue) and independent (yellow) sub‐secretomes indicated that proteins responsible for pro‐cardiovasculogenic functionality were largely contained within the miR‐146 dependent subset. Indeed, this sub‐secretome included 77%‐100% of proteins associated with individual enriched cardiovascular functions within the full secretome

Secretome upstream analysis projected 65 miRNAs as potential regulators (Figure 2A). Out of 447 miRNAs detected by next‐generation sequencing, 17 were differentially expressed (>1.5‐fold up or down, *P* < .05; Figure 2B upper) segregating high vs low response cardiopoietic cells (Figure 2B, lower). Among four miRNAs that overlapped between predicted (Figure 2C, blue) and observed (Figure 2C, red) regulators, only the miR‐146 family exhibited consistent directionality of expression change (Figure 2C, black ellipse) with most significant *P*‐value (Figure 2A) and greatest downregulation (Figure 2B). Notably, miR‐146a‐5p and miR‐146b‐5p share a seed sequence and common gene targets.^18^ The downregulated miR‐146 cluster linked to a 14‐protein directed network, characterized by activation of NFκB, STAT1/6 and CREB1 transcription pathways (Figure 3A upper). The miR‐146 dependent cassette was linked downstream with 101 of the 155 protein differential secretome (Figure 3A lower), yielding a 430 node extended neighborhood (Figure 3B; supporting Table S3). Accordingly, the miR‐146 dependent network encompassed enrichment consistent with prioritized cardio‐vasculogenesis of the full secretome (Figure 3C and Figure 3D; supporting Table S4). Conversely, the miR‐146 independent 54 proteins of the differential secretome lacked this signature (Figure 3D). Thus, systems interrogation specifies integration of miRNome and secretome functionality in high response cardiopoietic cells.

### 3.3 Favorable outcome reflects pro‐cardiovasculogenic impact

**FIGURE 4:**
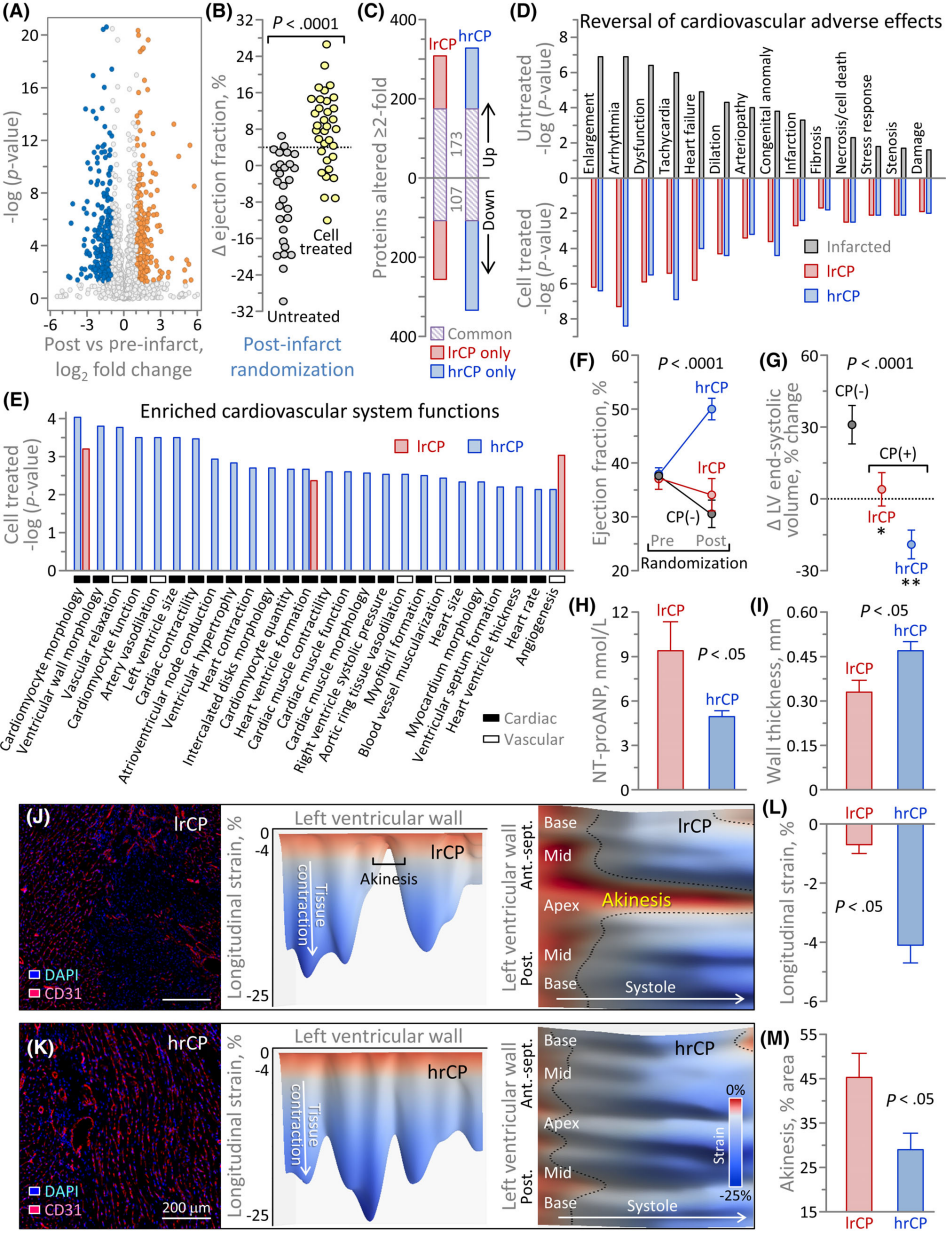
Subproteome linked to cardiopoietic cell therapeutic response. A, At 2‐months postinfarction, 13.7% of the murine ventricular proteome differed (6.4% up, 7.3% down ≥2‐fold, *P* < .05) compared with non‐infarction, measured by label‐free quantitative proteomics and shown by volcano plot. B, Ejection fraction on average decreased −7 ± 2% in untreated infarction (n = 28) but improved +8 ± 1% (n = 34; *P* < .0001) with human cardiopoietic cell (600 000 cells/heart delivered epicardially) treatment. C, Compared with untreated infarcted hearts, high and low response proteomes (hrCP and lrCP) exhibited a common core of 280 changes (purple), with a greater number of additional proteins altered in hrCP (blue) vs lrCP (red). D, Ingenuity Pathway Analysis prioritized infarction associated (top ranked shown, gray) cardiac adverse effects (*P* < .05) predicted to be nullified through full or partial subproteome reversal in both lrCP and hrCP cohorts. E, hrCP hearts were enriched across cardiac function, structure, conduction, muscularization and angiogenesis, while lrCP hearts displayed limited overlap in the predicted systems spectrum. hrCP achieved superior pump function (F), reversal of left ventricular (LV) enlargement (G), a decrease in the heart failure biomarker plasma NT‐proANP (H), and reduced wall thinning (I). Distinguishing from lrCP (J), hrCP infarcted myocardium expressed greater CD31+ staining with DAPI‐counterstained nuclei (K left), higher contractility (K middle) and decreased akinetic area (K right). Compared with lrCP, on average, hrCP hearts improved contractility in the infarcted region (L) and reduced the extent of akinetic scar (M)

In murine infarcted hearts with altered myocardial proteome (Figure 4A), delivery of human cardiopoietic cells improved cardiac performance (n = 34) in contrast to untreated counterparts (n = 28; Figure 4B). Compared with untreated, high (ΔEF >4%; hrCP) vs low (ΔEF <0%; lrCP) response treated hearts displayed greater proteome adjustments, with 154 proteins up and 227 down in hrCP vs 134 up and 151 down in lrCP, beyond the 280 proteins commonly altered (Figure 4C). Adverse outcomes of infarcted proteome were projected to be countered in hrCP and lrCP cohorts (Figure 4D). Notably, however, the hrCP reformed proteome was distinguished from lrCP by enhanced aptitude to engage across enriched cardiovascular system functions (Figure 4E). hrCP superiority was supported by documented improvement in cardiac pump function and reversal of chamber enlargement, with reductions in a heart failure biomarker and wall thinning (Figure 4F‐I), achieving reverse remodeling of ischemic cardiomyopathy. In hrCP relative to lrCP, regional mapping unveiled CD31+ tissue, greater contractility, and reduced akinetic scar,^14^ indicating myocardial viability (Figure 4J‐M). Thus, responsiveness of treated hearts reflected functionality inherent to the cardiopoietic cell secretome.

## 4 DISCUSSION

Heterogeneity in regenerative outcome is multifactorial.^19^, ^20^ While genetic and structural determinants intrinsic to recipient hearts are recognized, less is known regarding stem cell characteristics governing therapeutic effectiveness.^16^, ^21^, ^22^ We explored here, at systems level, cardiopoietic cell imprints that segregate with benefit. Merging multi‐omics datasets provides inclusive, unbiased strategies enabling functional prioritization of complex multidimensional outputs.^23^ Systems integration of a (cardio)vasculogenic secretome, arising from a distinct intracellular miRNA profile, distinguished cardiopoietic cells endowed with enhanced capacity. This is concordant with miRNA centrality in regulating regenerative and cardioprotective capacity.^24^, ^25^ Downregulation of miR‐146a‐5p or miR‐146b‐5p alters paracrine‐mediated immunomodulatory outcomes in cardiac signaling and facilitates repair postinfarction.^26^, ^27^ The present study supports the notion that secretome proficiency contributes to rescue of organ failure.^28^, ^29^, ^30^, ^31^, ^32^, ^33^, ^34^ Indeed, paracrine functionality was echoed in proteome restoration, underscoring connectivity between secretome signature and realized regenerative efficacy. Predelivery molecular profiling would thus aid in forecasting suitability of paracrine action. Moreover, assessment of the interaction of transplanted cells with recipient tissue, in conjunction with pharmaco‐kinetic/dynamic secretome behavior, would further advance the translational readiness of paracrine‐based biotherapy.

## 5 CONCLUSION

This proof‐of‐concept study suggests that therapeutic fitness is inherent to the cardiopoietic cell secretome. Pre‐intervention profiling would offer a predictive strategy to optimize cardioregenerative biologics, refined by understanding secretome fate postdelivery.

## CONFLICT OF INTEREST

R.J.C.D., S.Y., A.B., and A.T. are co‐inventors on regenerative sciences related intellectual property disclosed to Mayo Clinic. Mayo Clinic administered previous research grants from Celyad. Mayo Clinic, A.B., and A.T. have interests in Rion LLC.

## AUTHOR CONTRIBUTIONS

D.K.A.: conception and design, collection and assembly of data, data analysis and interpretation, manuscript writing, final approval of manuscript; R.J.C.D.: conception and design, collection and assembly of data, data analysis and interpretation, final approval of manuscript; S.Y.: conception and design, financial support, collection and assembly of data, data analysis and interpretation, manuscript writing, final approval of manuscript; R.J., A.G., S.P., J.P.A., C.L., M.L.H.: collection and assembly of data, data analysis and interpretation, final approval of manuscript; J.B.: provision of study material, data interpretation, final approval of manuscript; A.B.: conception and design, financial support, administrative support, provision of study material, data interpretation, final approval of manuscript; A.T.: conception and design, financial support, administrative support, provision of study material, data analysis and interpretation, manuscript writing, final approval of manuscript.

## Supporting information

**Appendix S1**: Supporting Information.

## Data Availability

The authors declare that all data supporting the findings of this study are available within the article and its Supporting Information files.
