# Supplementary material for: Secretome signature of cardiopoietic cells echoed in rescued infarcted heart proteome
Source: Stem Cells Transl Med. 2021 May 28;10(9):1320–8. doi: 10.1002/sctm.20-0509 (PMC8380441; doi:10.1002/sctm.20-0509)
Supplement: Supplementary file 1 — Appendix S1: Supporting Information. [file SCT3-10-1320-s001.pdf]

SUPPORTING INFORMATION

Secretome Signature of Cardiopoietic Cells Echoed in Rescued Infarcted Heart Proteome

D. Kent Arrell | Ruben J. Crespo-Diaz | Satsuki Yamada | Ryounghoon Jeon | Armin Garmany | Sungjo Park | Jeffrey P. Adolf | Christopher Livia | Matthew L. Hillestad | Jozef Bartunek | Atta Behfar | Andre Terzic |

SUPPORTING TABLES

Table S1. Differential secretome network nodes and topological properties. Using a 2-fold differential expression cutoff for upregulated (orange) or downregulated (blue) proteins, the measured secretome was submitted to Ingenuity Pathway Analysis (IPA) for network generation. Additional network-integrated (rose) nodes were derived from the IPA-based network neighborhood, obtained from direct and indirect relationships for derivation of pairwise interactions (edges). Network edges were undirected, unweighted, with multi-edge node pairs merged as a single interaction, yielding a 520 node network comprised of 8977 edges, with connectivity exhibiting a power law distribution ($y = 46.50x^{-0.73}$, $r = 0.77$) indicative of non-stochastic scale-free architecture [1,2]. Imported into Cytoscape v.3.8.2 [3], the embedded application NetworkAnalyzer [4] was used to interpret network node topology. The node topological values for degree, clustering coefficient, average shortest path length, and betweenness centrality are included [1,2]. Degree represents number of connections, or edges, between a node and other nodes in the network. Clustering coefficient identifies the ratio of nodes connected to it, i.e. first neighbors, that also connect to one another, relative to total number of possible edges between first neighbors of the node. Average shortest path length

represents the mean path length to all other nodes in the network, where the length of each node pair equals the minimum number of edges required to connect the nodes. Betweenness centrality measures the proportion of all shortest paths within the network that pass through that node.

Table S1 – Differential secretome network nodes and topological properties

Node	Network Inclusion Basis	Log_2 FC	Degree	Clustering Coefficient	Average Shortest Path Length	Betweenness Centrality
ACKR2	Upregulated in Secretome	1.196	15	0.476	2.102	2.76E-04
ACVR1	Upregulated in Secretome	3.169	23	0.271	2.262	0.00137026
ACVR1B	Upregulated in Secretome	1.539	8	0.357	2.898	1.14E-04
ACVR2B	Upregulated in Secretome	1.063	18	0.248	2.231	0.00111215
AGER	Upregulated in Secretome	1.431	51	0.441	1.954	0.00163980
AGRP	Upregulated in Secretome	1.193	6	0.667	2.422	3.15E-06
ALCAM	Upregulated in Secretome	10.46	25	0.451	2.052	1.08E-04
ANGPT1	Upregulated in Secretome	1.107	33	0.639	2.012	1.17E-04
AREG	Upregulated in Secretome	1.017	49	0.391	1.979	0.00110732
BIK	Upregulated in Secretome	8.153	11	0.345	2.222	1.60E-04
BMP5	Upregulated in Secretome	1.810	5	0.300	2.505	1.99E-04
BPIFA1	Upregulated in Secretome	1.881	4	0.833	2.370	3.20E-07
CCL16	Upregulated in Secretome	1.031	5	0	2.676	1.06E-05
CCL20	Upregulated in Secretome	1.023	50	0.367	1.958	0.00125906
CCL21	Upregulated in Secretome	1.380	19	0.456	2.114	2.10E-04
CCL25	Upregulated in Secretome	1.450	13	0.385	2.270	7.32E-05
CCL7	Upregulated in Secretome	1.567	30	0.460	2.023	4.50E-04
CCN3	Upregulated in Secretome	1.011	22	0.416	2.067	4.00E-04
CD14	Upregulated in Secretome	1.404	61	0.375	1.944	0.00188661
CD163	Upregulated in Secretome	2.891	14	0.409	2.175	1.46E-04
CD40	Upregulated in Secretome	1.213	113	0.239	1.823	0.01255221
CD40LG	Upregulated in Secretome	1.157	102	0.255	1.854	0.00740950
CD80	Upregulated in Secretome	3.153	54	0.416	1.981	0.00147156
CER1	Upregulated in Secretome	1.179	3	0.333	2.740	2.23E-05
CRIM1	Upregulated in Secretome	1.340	1	0	2.805	0
CSF2RA	Upregulated in Secretome	1.025	12	0.178	2.331	8.48E-05
CTNNB1	Upregulated in Secretome	1.593	135	0.201	1.761	0.02373687
CXCL13	Upregulated in Secretome	1.921	25	0.387	2.060	3.81E-04
CXCL2	Upregulated in Secretome	1.837	64	0.269	1.929	0.00318728
CXCR3	Upregulated in Secretome	1.060	34	0.426	2.015	4.45E-04
DEFB1	Upregulated in Secretome	10.82	12	0.106	2.563	1.79E-04
DEFB4A/DEFB4B	Upregulated in Secretome	7.654	32	0.485	2.054	4.66E-04
DKK1	Upregulated in Secretome	2.001	25	0.310	2.064	0.00126210
EDAR	Upregulated in Secretome	1.067	6	0.800	2.222	1.51E-06
EGF	Upregulated in Secretome	1.040	160	0.184	1.711	0.02409543
EGFR	Upregulated in Secretome	1.124	179	0.187	1.672	0.02768148

FADD	Upregulated in Secretome	1.010	34	0.444	2.031	4.75E-04
FGF6	Upregulated in Secretome	1.279	7	0.476	2.428	9.55E-06
FLT3	Upregulated in Secretome	1.067	26	0.475	2.067	2.19E-04
FRZB	Upregulated in Secretome	1.019	9	0.190	2.272	3.68E-04
FSTL3	Upregulated in Secretome	1.090	10	0.422	2.347	1.94E-04
FZD1	Upregulated in Secretome	2.647	14	0.348	2.220	1.22E-04
FZD3	Upregulated in Secretome	1.126	5	0.600	2.530	7.01E-06
FZD7	Upregulated in Secretome	1.011	4	0.167	2.628	8.90E-06
GDF1	Upregulated in Secretome	1.059	1	0	3.104	0
GDF10	Upregulated in Secretome	1.402	3	0	2.728	9.75E-06
GDF5	Upregulated in Secretome	1.040	15	0.141	2.653	5.25E-04
GFRA3	Upregulated in Secretome	1.538	1	0	3.125	0
GH1	Upregulated in Secretome	2.300	37	0.370	2.048	6.21E-04
HCRTR1	Upregulated in Secretome	1.220	5	0.500	2.366	2.30E-05
HGF	Upregulated in Secretome	1.091	109	0.242	1.815	0.01051231
HRG	Upregulated in Secretome	2.321	7	0.286	2.276	5.78E-05
IGF1R	Upregulated in Secretome	1.249	91	0.285	1.857	0.00556286
IGFBP3	Upregulated in Secretome	1.050	57	0.358	1.933	0.00314263
IGFBP6	Upregulated in Secretome	1.134	14	0.374	2.135	3.91E-04
IGFBP7	Upregulated in Secretome	1.701	15	0.390	2.175	2.51E-04
IL13RA1	Upregulated in Secretome	1.889	12	0.409	2.407	4.88E-05
IL17RA	Upregulated in Secretome	1.134	24	0.312	2.125	0.00124729
IL17RB	Upregulated in Secretome	2.584	14	0.505	2.116	9.25E-04
IL17RD	Upregulated in Secretome	1.071	10	0.756	2.133	2.71E-05
IL18R1	Upregulated in Secretome	2.067	25	0.553	2.100	2.57E-04
IL1B	Upregulated in Secretome	1.095	249	0.142	1.536	0.05581781
IL1R2	Upregulated in Secretome	1.121	21	0.386	2.108	4.59E-04
IL21R	Upregulated in Secretome	1.708	19	0.412	2.225	1.01E-04
IL2RA	Upregulated in Secretome	2.572	58	0.411	1.950	0.00120549
IL5RA	Upregulated in Secretome	1.208	15	0.372	2.349	9.12E-05
IL7R	Upregulated in Secretome	1.805	32	0.419	2.033	0.00110582
IL9	Upregulated in Secretome	1.777	53	0.280	1.973	0.00259936
INHBB	Upregulated in Secretome	1.139	22	0.342	2.133	0.00146804
ITGAL	Upregulated in Secretome	1.051	33	0.460	2.013	4.73E-04
LCK	Upregulated in Secretome	2.147	66	0.331	1.938	0.00298968
LECT2	Upregulated in Secretome	1.528	7	0.500	2.231	1.47E-04
LEFTY2	Upregulated in Secretome	2.370	4	0.333	2.495	1.98E-04
LEP	Upregulated in Secretome	2.369	105	0.249	1.829	0.00878198
LTBP1	Upregulated in Secretome	2.009	14	0.505	2.135	6.08E-05
MFRP	Upregulated in Secretome	1.297	1	0	3.829	0
MICOS10-NBL1/NBL1	Upregulated in Secretome	1.801	5	0.100	2.705	4.90E-05
MMP11	Upregulated in Secretome	1.669	17	0.305	2.189	4.38E-04
MMP24	Upregulated in Secretome	1.248	4	0.167	2.807	2.36E-05
MMP25	Upregulated in Secretome	2.494	10	0.067	2.441	2.70E-04
MMP9	Upregulated in Secretome	1.346	151	0.220	1.728	0.02442419
NGF	Upregulated in Secretome	1.427	83	0.278	1.882	0.00551956
NRG1	Upregulated in Secretome	2.597	69	0.280	1.929	0.00746085
NRG2	Upregulated in Secretome	2.291	13	0.167	2.270	6.23E-04
NTF4	Upregulated in Secretome	1.046	7	0.400	2.445	5.75E-06
PDGFA	Upregulated in Secretome	1.971	34	0.363	2.019	0.00102798

PF4	Upregulated in Secretome	2.252	39	0.402	2.015	0.00151662
PGF	Upregulated in Secretome	1.158	25	0.538	2.042	4.79E-04
PRL	Upregulated in Secretome	1.093	78	0.323	1.879	0.00622396
SELE	Upregulated in Secretome	1.797	65	0.331	1.919	0.00347760
SELP	Upregulated in Secretome	1.292	42	0.354	2.029	8.63E-04
SFRP4	Upregulated in Secretome	1.480	6	0.200	2.611	1.14E-04
SMAD1	Upregulated in Secretome	3.058	41	0.248	1.990	0.00345762
SMAD9	Upregulated in Secretome	2.324	8	0.467	2.308	6.91E-05
TFPI	Upregulated in Secretome	1.073	14	0.485	2.160	3.85E-05
TLR3	Upregulated in Secretome	1.276	63	0.361	1.969	0.00276959
TLR4	Upregulated in Secretome	1.350	124	0.247	1.802	0.01053974
TNFAIP6	Upregulated in Secretome	1.049	32	0.331	2.015	0.00166898
TNFRSF10A	Upregulated in Secretome	1.719	26	0.496	2.040	5.31E-04
TNFRSF12A	Upregulated in Secretome	2.470	20	0.301	2.150	2.65E-04
TNFRSF17	Upregulated in Secretome	4.737	10	0.711	2.291	1.45E-05
TNFRSF19	Upregulated in Secretome	1.331	6	0.500	2.434	1.19E-05
TNFRSF25	Upregulated in Secretome	2.430	23	0.443	2.114	3.11E-04
TNFRSF6B	Upregulated in Secretome	1.005	11	0.722	2.170	5.46E-06
TNFSF11	Upregulated in Secretome	1.724	82	0.360	1.869	0.00631266
TNFSF18	Upregulated in Secretome	1.055	10	0.489	2.318	4.12E-05
TYMP	Upregulated in Secretome	1.452	18	0.742	2.079	1.32E-05
26s Proteasome	Network-integrated by IPA		46	0.283	1.983	0.00402958
7S NGF	Network-integrated by IPA		6	0.600	2.247	7.65E-06
AChR	Network-integrated by IPA		13	0.345	2.143	4.73E-05
Actin	Network-integrated by IPA		58	0.266	1.963	0.00254670
Activin	Network-integrated by IPA		6	0.533	2.644	2.30E-05
ADCY	Network-integrated by IPA		42	0.386	1.979	0.00112474
adhesion molecule	Network-integrated by IPA		15	0.686	2.114	6.40E-05
ADRB	Network-integrated by IPA		25	0.415	2.100	2.02E-04
Akt	Network-integrated by IPA		231	0.162	1.566	0.04584410
ALDH	Network-integrated by IPA		4	0.833	2.254	1.21E-06
Alp	Network-integrated by IPA		57	0.299	1.931	0.00886858
Alpha 1 antitrypsin	Network-integrated by IPA		5	0.500	2.445	3.15E-05
Alpha Actinin	Network-integrated by IPA		18	0.433	2.239	4.12E-05
Alpha catenin	Network-integrated by IPA		36	0.326	2.010	6.80E-04
ALT	Network-integrated by IPA		12	0.439	2.175	1.41E-04
Ampa Receptor	Network-integrated by IPA		21	0.509	2.108	6.36E-05
AMPK	Network-integrated by IPA		65	0.323	1.911	0.00615539
amylase	Network-integrated by IPA		4	0.333	2.568	1.64E-06
Angiotensin II receptor type 1	Network-integrated by IPA		16	0.604	2.129	2.40E-05
Ap1	Network-integrated by IPA		108	0.307	1.821	0.00578316
Ap2	Network-integrated by IPA		8	0.679	2.202	5.68E-06
Atrial Natriuretic Peptide	Network-integrated by IPA		10	0.444	2.277	4.77E-05
BCR (complex)	Network-integrated by IPA		74	0.270	1.927	0.00305543
BMP	Network-integrated by IPA		9	0.278	2.453	3.65E-04
C/EBP	Network-integrated by IPA		25	0.628	2.046	7.26E-05
C1q	Network-integrated by IPA		23	0.481	2.087	1.51E-04
C1QTNF5	Network-integrated by IPA		5	0	2.830	0.00385410
C8	Network-integrated by IPA		4	0.500	2.474	2.12E-06

CALC	Network-integrated by IPA	17	0.667	2.100	1.41E-04
Calcineurin A	Network-integrated by IPA	17	0.610	2.083	5.44E-05
Calcineurin protein(s)	Network-integrated by IPA	40	0.387	2.017	7.20E-04
Calmodulin	Network-integrated by IPA	52	0.331	1.971	0.00259892
calpain	Network-integrated by IPA	44	0.370	1.996	8.70E-04
CaMKII	Network-integrated by IPA	42	0.397	2.029	6.09E-04
cAMP-dependent protein kinase	Network-integrated by IPA	14	0.212	2.224	1.23E-04
Casein	Network-integrated by IPA	10	0.679	2.173	8.47E-06
caspase	Network-integrated by IPA	61	0.319	1.944	0.00169845
Caspase 3/7	Network-integrated by IPA	15	0.419	2.139	9.58E-05
Cbp/p300	Network-integrated by IPA	24	0.286	2.064	6.88E-04
C-C chemokine receptor	Network-integrated by IPA	14	0.303	2.468	4.81E-05
CCR6	Network-integrated by IPA	24	0.391	2.104	5.55E-04
CD3	Network-integrated by IPA	91	0.257	1.882	0.00462799
CD3 group	Network-integrated by IPA	18	0.588	2.098	3.47E-05
CD4	Network-integrated by IPA	44	0.358	2.002	7.49E-04
CD80/CD86	Network-integrated by IPA	5	0.600	2.291	1.10E-05
CD81	Network-integrated by IPA	34	0.365	2.054	3.84E-04
CD9	Network-integrated by IPA	27	0.497	2.054	1.41E-04
Cebp	Network-integrated by IPA	6	0.600	2.227	5.85E-05
CG	Network-integrated by IPA	43	0.416	1.967	0.00226555
chemokine	Network-integrated by IPA	54	0.278	2.002	0.00269833
chemokine receptor	Network-integrated by IPA	13	0.273	2.453	4.47E-05
Ciap	Network-integrated by IPA	12	0.500	2.141	3.19E-05
Ck2	Network-integrated by IPA	29	0.296	2.050	4.76E-04
Cofilin	Network-integrated by IPA	33	0.454	2.023	2.76E-04
collagen	Network-integrated by IPA	27	0.490	2.031	1.57E-04
Collagen Alpha1	Network-integrated by IPA	30	0.495	2.044	3.67E-04
Collagen type I (complex)	Network-integrated by IPA	46	0.455	1.960	0.00100575
collagen type I (family)	Network-integrated by IPA	30	0.354	2.013	0.00124740
Collagen type II	Network-integrated by IPA	19	0.520	2.077	4.49E-04
Collagen type III	Network-integrated by IPA	8	0.821	2.227	3.13E-06
Collagen type IV	Network-integrated by IPA	31	0.346	2.052	3.87E-04
Collagen(s)	Network-integrated by IPA	64	0.374	1.921	0.00152763
Complement	Network-integrated by IPA	20	0.490	2.137	6.34E-05
Cpla2	Network-integrated by IPA	22	0.606	2.062	4.63E-05
CPT1	Network-integrated by IPA	8	0.267	2.721	1.09E-05
Cr3	Network-integrated by IPA	21	0.497	2.100	6.11E-05
Creb	Network-integrated by IPA	92	0.302	1.846	0.00495748
c-Src	Network-integrated by IPA	11	0.583	2.252	5.28E-06
Cyclin A	Network-integrated by IPA	38	0.437	1.979	3.46E-04
Cyclin B	Network-integrated by IPA	12	0.439	2.266	2.28E-05
Cyclin D	Network-integrated by IPA	33	0.465	2.021	7.60E-04
Cyclin E	Network-integrated by IPA	25	0.597	2.033	1.12E-04
cyclooxygenase	Network-integrated by IPA	14	0.615	2.096	1.07E-04
CYP19	Network-integrated by IPA	10	0.750	2.141	6.22E-06
cytochrome C	Network-integrated by IPA	30	0.421	2.050	2.98E-04

cytokine	Network-integrated by IPA	138	0.223	1.765	0.01417424
cytokine receptor	Network-integrated by IPA	27	0.170	2.277	4.36E-04
death receptor	Network-integrated by IPA	7	0.714	2.353	5.21E-06
DEFB103A/DEFB103B	Network-integrated by IPA	27	0.497	2.064	5.53E-04
DNA-PK	Network-integrated by IPA	7	0.500	2.337	4.11E-06
Dynamin	Network-integrated by IPA	17	0.706	2.087	3.92E-05
E2f	Network-integrated by IPA	19	0.537	2.094	1.56E-04
Ecm	Network-integrated by IPA	12	0.500	2.262	2.50E-05
EF-1 alpha	Network-integrated by IPA	11	0.509	2.287	1.62E-05
EGFR ligand	Network-integrated by IPA	9	0.639	2.432	1.23E-05
EGFR/PDGFR/IGFR	Network-integrated by IPA	7	0.810	2.356	3.59E-06
EGLN	Network-integrated by IPA	6	0.133	2.620	3.97E-05
elastase	Network-integrated by IPA	17	0.574	2.096	1.13E-04
Eotaxin	Network-integrated by IPA	9	0.639	2.175	1.05E-05
ERBB	Network-integrated by IPA	16	0.383	2.314	9.68E-05
Erbb1 dimer	Network-integrated by IPA	7	0.619	2.466	7.39E-06
Erbb1-Erbb4 dimer	Network-integrated by IPA	3	1.000	2.632	0
Erbb4 dimer	Network-integrated by IPA	8	0.429	2.516	7.43E-05
ERBB4 ligand	Network-integrated by IPA	5	0.500	2.798	7.13E-06
ERK	Network-integrated by IPA	198	0.189	1.630	0.02257525
ERK1/2	Network-integrated by IPA	253	0.146	1.522	0.05730879
Erm	Network-integrated by IPA	23	0.443	2.114	1.61E-04
estrogen receptor	Network-integrated by IPA	55	0.307	1.942	0.00687069
ETS	Network-integrated by IPA	11	0.618	2.112	6.78E-05
EZR	Network-integrated by IPA	53	0.392	1.956	9.22E-04
F Actin	Network-integrated by IPA	48	0.298	2.004	0.00112561
farnesyl transferase	Network-integrated by IPA	9	0.833	2.152	2.27E-06
Fascin	Network-integrated by IPA	14	0.667	2.104	2.31E-05
Fc gamma receptor	Network-integrated by IPA	35	0.469	2.012	3.63E-04
Fcer1	Network-integrated by IPA	41	0.454	1.992	5.89E-04
Fcgr2	Network-integrated by IPA	9	0.667	2.191	6.89E-06
Fcgr3	Network-integrated by IPA	22	0.498	2.066	1.66E-04
Fgf	Network-integrated by IPA	34	0.335	2.048	0.00129124
Fgfr	Network-integrated by IPA	28	0.474	2.091	6.50E-04
Fibrin	Network-integrated by IPA	15	0.448	2.204	1.13E-04
Fibrinogen	Network-integrated by IPA	38	0.381	2.002	0.00109532
Filamin	Network-integrated by IPA	13	0.410	2.224	3.10E-05
FKHR	Network-integrated by IPA	5	0.700	2.362	2.00E-06
Focal adhesion kinase	Network-integrated by IPA	63	0.416	1.931	0.00124524
Foxo	Network-integrated by IPA	12	0.489	2.164	1.28E-04
Frizzled	Network-integrated by IPA	14	0.318	2.401	3.89E-04
FSH	Network-integrated by IPA	54	0.300	1.954	0.00446416
G protein	Network-integrated by IPA	45	0.280	2.035	0.00143373
G protein alphai	Network-integrated by IPA	31	0.522	2.066	1.66E-04
G protein beta gamma	Network-integrated by IPA	26	0.566	2.098	1.56E-04
GABP	Network-integrated by IPA	4	0	2.765	7.28E-06
Gap	Network-integrated by IPA	15	0.705	2.216	1.41E-05
GC-GCR dimer	Network-integrated by IPA	8	0.214	2.337	4.04E-05
Gcn5l	Network-integrated by IPA	7	0.238	2.672	3.11E-05
gelatinase	Network-integrated by IPA	12	0.818	2.119	6.05E-06

GLP2R	Network-integrated by IPA	5	0.667	2.476	9.30E-07
Glycogen synthase	Network-integrated by IPA	19	0.544	2.125	5.94E-05
GNRH	Network-integrated by IPA	23	0.514	2.106	0.00391951
GOT	Network-integrated by IPA	10	0.667	2.150	1.82E-05
Gpcr	Network-integrated by IPA	57	0.298	1.996	0.00379348
G-protein beta	Network-integrated by IPA	24	0.481	2.183	1.20E-04
growth factor	Network-integrated by IPA	32	0.587	2.002	2.42E-04
growth factor receptor	Network-integrated by IPA	24	0.450	2.189	3.15E-04
Growth hormone	Network-integrated by IPA	60	0.289	1.934	0.00323770
Gsk3	Network-integrated by IPA	57	0.389	1.925	0.00143901
Hdac	Network-integrated by IPA	35	0.242	2.029	0.00119344
HDL	Network-integrated by IPA	41	0.503	1.988	4.65E-04
hemoglobin	Network-integrated by IPA	14	0.318	2.177	8.41E-04
Hif1	Network-integrated by IPA	28	0.329	2.094	2.31E-04
HISTONE	Network-integrated by IPA	25	0.372	2.062	5.55E-04
histone deacetylase	Network-integrated by IPA	17	0.272	2.114	3.41E-04
Histone h3	Network-integrated by IPA	74	0.354	1.888	0.00298870
Histone h4	Network-integrated by IPA	44	0.328	1.975	0.00156388
HLA-DR	Network-integrated by IPA	24	0.551	2.079	1.23E-04
HLA-DR group	Network-integrated by IPA	13	0.718	2.106	2.02E-05
HRAS	Network-integrated by IPA	112	0.266	1.807	0.01224617
Hsp27	Network-integrated by IPA	47	0.447	1.971	0.00122219
Hsp70	Network-integrated by IPA	52	0.422	1.929	9.21E-04
Hsp90	Network-integrated by IPA	60	0.391	1.925	0.00102522
Hspg	Network-integrated by IPA	12	0.424	2.262	7.96E-05
I kappa b kinase	Network-integrated by IPA	20	0.579	2.067	1.71E-04
Icam	Network-integrated by IPA	7	0.333	2.274	4.68E-05
Ifn	Network-integrated by IPA	49	0.363	1.988	7.78E-04
IFN alpha/beta	Network-integrated by IPA	19	0.478	2.222	1.32E-04
IFN Beta	Network-integrated by IPA	61	0.418	1.934	0.00122841
Ifn gamma	Network-integrated by IPA	32	0.502	2.021	2.15E-04
IFN type 1	Network-integrated by IPA	19	0.427	2.243	1.07E-04
Ifnar	Network-integrated by IPA	16	0.575	2.171	8.38E-05
Iga	Network-integrated by IPA	37	0.415	2.042	7.08E-04
IgD	Network-integrated by IPA	9	0.810	2.514	1.12E-06
Ige	Network-integrated by IPA	76	0.304	1.906	0.00343837
Igf	Network-integrated by IPA	14	0.637	2.119	2.00E-04
Igfbp	Network-integrated by IPA	11	0.164	2.489	7.18E-05
IgG	Network-integrated by IPA	93	0.256	1.859	0.00720870
IgG1	Network-integrated by IPA	35	0.368	2.050	8.41E-04
IgG2a	Network-integrated by IPA	29	0.426	2.066	4.20E-04
IgG2b	Network-integrated by IPA	13	0.538	2.204	3.78E-05
IgG2c	Network-integrated by IPA	3	0.333	2.784	2.03E-06
Igg3	Network-integrated by IPA	20	0.411	2.187	1.58E-04
IgG4	Network-integrated by IPA	7	0.571	2.341	2.39E-05
Igm	Network-integrated by IPA	64	0.355	1.950	0.00185718
Ikb	Network-integrated by IPA	40	0.422	2.015	5.79E-04
IKK (complex)	Network-integrated by IPA	46	0.443	1.944	7.38E-04
Ikk (family)	Network-integrated by IPA	28	0.538	2.058	2.44E-04
IL1	Network-integrated by IPA	139	0.233	1.751	0.01247602

IL12 (complex)	Network-integrated by IPA	96	0.294	1.859	0.00416195
IL12 (family)	Network-integrated by IPA	79	0.310	1.911	0.00285286
IL12RB2/IL23R	Network-integrated by IPA	4	1.000	2.299	0
IL17a dimer	Network-integrated by IPA	12	0.470	2.195	1.17E-04
IL-17f dimer	Network-integrated by IPA	8	0.250	2.447	5.70E-05
IL17R	Network-integrated by IPA	18	0.346	2.185	2.88E-04
IL-1R	Network-integrated by IPA	26	0.543	2.062	1.20E-04
IL-1R/TLR	Network-integrated by IPA	5	0.700	2.620	2.77E-06
IL23	Network-integrated by IPA	32	0.466	2.056	5.36E-04
IL-2R	Network-integrated by IPA	19	0.444	2.233	1.47E-04
IL31	Network-integrated by IPA	20	0.642	2.058	2.68E-04
Il8r	Network-integrated by IPA	17	0.596	2.083	5.72E-05
Immunoglobulin	Network-integrated by IPA	134	0.220	1.775	0.01148931
Importin alpha	Network-integrated by IPA	9	0.583	2.239	1.11E-05
Inhibin	Network-integrated by IPA	6	0.533	2.397	2.14E-05
Insulin	Network-integrated by IPA	118	0.231	1.798	0.01084434
Integrin	Network-integrated by IPA	74	0.300	1.908	0.00326942
Integrin alpha V beta 3	Network-integrated by IPA	14	0.352	2.260	3.78E-05
Interferon alpha	Network-integrated by IPA	87	0.308	1.873	0.00408189
INTERLEUKIN	Network-integrated by IPA	4	0.333	2.923	8.78E-06
IRAK	Network-integrated by IPA	20	0.562	2.141	7.91E-05
IRF	Network-integrated by IPA	16	0.517	2.193	1.05E-04
JAK	Network-integrated by IPA	36	0.351	2.067	6.08E-04
JAK1/2	Network-integrated by IPA	7	0.381	2.235	2.83E-05
JINK1/2	Network-integrated by IPA	32	0.402	2.019	3.52E-04
Jnk	Network-integrated by IPA	196	0.180	1.634	0.02601904
JUN/JUNB/JUND	Network-integrated by IPA	21	0.398	2.100	4.12E-04
L1CAM	Network-integrated by IPA	27	0.457	2.050	2.38E-04
Laminin (complex)	Network-integrated by IPA	45	0.405	1.960	0.00110584
Laminin1	Network-integrated by IPA	16	0.633	2.094	2.77E-05
Ldh (complex)	Network-integrated by IPA	21	0.462	2.092	1.30E-04
LDL	Network-integrated by IPA	81	0.360	1.873	0.00295709
LDL-cholesterol	Network-integrated by IPA	6	0.333	2.408	2.74E-05
Lfa-1	Network-integrated by IPA	15	0.295	2.397	3.80E-05
LGALS1	Network-integrated by IPA	41	0.328	1.998	0.00141695
LGALS3	Network-integrated by IPA	45	0.385	1.963	8.07E-04
Lh	Network-integrated by IPA	51	0.410	1.948	0.00170309
Lymphotoxin	Network-integrated by IPA	10	0.556	2.204	4.09E-05
MAC	Network-integrated by IPA	18	0.542	2.089	2.72E-04
MAP2K1/2	Network-integrated by IPA	64	0.389	1.911	0.00140758
Mapk	Network-integrated by IPA	157	0.209	1.715	0.01652644
MEF2	Network-integrated by IPA	15	0.390	2.133	7.51E-05
Mek	Network-integrated by IPA	75	0.397	1.890	0.00189858
Metalloprotease	Network-integrated by IPA	28	0.413	2.025	0.00144494
MHC Class I (complex)	Network-integrated by IPA	17	0.404	2.177	2.22E-04
MHC CLASS I (family)	Network-integrated by IPA	20	0.425	2.114	1.42E-04
MHC Class II (complex)	Network-integrated by IPA	36	0.426	2.050	5.61E-04
MHC II	Network-integrated by IPA	22	0.558	2.069	1.67E-04
mir-34	Network-integrated by IPA	17	0.457	2.127	0.00389202

MITF	Network-integrated by IPA	30	0.508	2.031	8.68E-04
MKK3/6	Network-integrated by IPA	16	0.558	2.096	8.14E-05
Mlc	Network-integrated by IPA	35	0.360	2.050	4.54E-04
Mmp	Network-integrated by IPA	63	0.352	1.923	0.00397411
Mt-mmp	Network-integrated by IPA	5	0.100	2.696	6.68E-05
MTORC1	Network-integrated by IPA	35	0.413	2.012	0.00129915
Mucin	Network-integrated by IPA	14	0.253	2.235	9.56E-05
myosin-light-chain kinase	Network-integrated by IPA	19	0.632	2.096	4.19E-05
Na+,K+ -ATPase	Network-integrated by IPA	17	0.500	2.091	1.06E-04
NADPH oxidase	Network-integrated by IPA	37	0.561	2.000	2.31E-04
N-Cadherin	Network-integrated by IPA	8	0.857	2.206	2.28E-06
NCK	Network-integrated by IPA	22	0.505	2.175	1.52E-04
N-cor	Network-integrated by IPA	24	0.322	2.054	6.51E-04
Neurotrophin	Network-integrated by IPA	20	0.563	2.094	3.82E-04
NFAT (complex)	Network-integrated by IPA	24	0.420	2.133	1.67E-04
Nfat (family)	Network-integrated by IPA	55	0.398	1.967	9.06E-04
NFkB (complex)	Network-integrated by IPA	222	0.163	1.584	0.04253958
NFkB (family)	Network-integrated by IPA	24	0.571	2.033	1.51E-04
NfkB1-RelA	Network-integrated by IPA	18	0.575	2.098	7.53E-05
NfkB-RelA	Network-integrated by IPA	10	0.643	2.222	8.50E-06
Ngf	Network-integrated by IPA	7	0.095	2.671	4.02E-05
NMT1	Network-integrated by IPA	4	0.333	2.318	4.65E-05
NMT2	Network-integrated by IPA	5	0.400	2.299	5.38E-05
Nos	Network-integrated by IPA	37	0.492	1.998	9.23E-04
Notch	Network-integrated by IPA	25	0.458	2.071	1.24E-04
Nr1h	Network-integrated by IPA	26	0.351	2.092	6.18E-04
NRG (family)	Network-integrated by IPA	22	0.479	2.108	4.89E-04
P glycoprotein	Network-integrated by IPA	24	0.459	2.075	1.75E-04
P38 MAPK	Network-integrated by IPA	203	0.183	1.617	0.02835993
p70 S6k	Network-integrated by IPA	49	0.499	1.961	5.90E-04
p85 (pik3r)	Network-integrated by IPA	51	0.455	1.963	5.74E-04
Pak	Network-integrated by IPA	29	0.504	2.064	1.84E-04
PARP	Network-integrated by IPA	21	0.476	2.083	6.56E-05
Pde4	Network-integrated by IPA	11	0.673	2.139	2.18E-05
Pdgf (complex)	Network-integrated by IPA	65	0.320	1.960	0.00224984
PDGF (family)	Network-integrated by IPA	9	0.444	2.281	1.69E-05
Pdgf Ab	Network-integrated by IPA	19	0.462	2.121	8.46E-05
PDGF BB	Network-integrated by IPA	71	0.310	1.931	0.00355671
PDGF-AA	Network-integrated by IPA	18	0.680	2.121	3.42E-05
PDGF-DD	Network-integrated by IPA	8	0.267	2.489	2.88E-05
Pdgfr	Network-integrated by IPA	46	0.426	2.037	5.48E-04
PEPCK	Network-integrated by IPA	23	0.245	2.193	4.03E-04
peptidase	Network-integrated by IPA	5	0.900	2.168	3.50E-07
PFK	Network-integrated by IPA	9	0.361	2.355	3.79E-05
PI3K (complex)	Network-integrated by IPA	201	0.187	1.624	0.02842886
PI3K (family)	Network-integrated by IPA	44	0.414	1.990	8.45E-04
PI3K beta	Network-integrated by IPA	14	0.571	2.114	8.59E-05
PI3K p85	Network-integrated by IPA	37	0.392	2.023	7.04E-04
PIK3CA	Network-integrated by IPA	54	0.363	1.950	0.00222445
Pka	Network-integrated by IPA	109	0.251	1.825	0.00763009

Pka catalytic subunit	Network-integrated by IPA	20	0.438	2.127	1.43E-04
Pkc(s)	Network-integrated by IPA	144	0.223	1.742	0.01402139
PLA2	Network-integrated by IPA	23	0.565	2.044	9.65E-05
PLC	Network-integrated by IPA	46	0.315	1.981	0.00135967
PLC gamma	Network-integrated by IPA	53	0.302	1.987	0.00197737
Pld	Network-integrated by IPA	47	0.396	1.971	6.90E-04
potassium channel	Network-integrated by IPA	7	0.667	2.303	3.32E-06
PP1 protein complex group	Network-integrated by IPA	36	0.385	2.054	6.63E-04
PP2A	Network-integrated by IPA	50	0.419	1.967	6.97E-04
Ppp2c	Network-integrated by IPA	19	0.566	2.112	5.16E-05
PRKAA	Network-integrated by IPA	26	0.508	2.050	8.05E-04
Pro-inflammatory Cytokine	Network-integrated by IPA	62	0.345	1.933	0.00200216
Proinsulin	Network-integrated by IPA	25	0.353	2.077	5.26E-04
PROTEASE	Network-integrated by IPA	10	0.667	2.152	1.42E-05
protein phosphatase	Network-integrated by IPA	10	0.622	2.125	1.99E-05
PTK	Network-integrated by IPA	23	0.486	2.079	1.46E-04
pyruvate kinase	Network-integrated by IPA	12	0.333	2.277	6.19E-05
Rab5	Network-integrated by IPA	11	0.600	2.145	2.32E-05
Rac	Network-integrated by IPA	75	0.331	1.902	0.00221807
Raf	Network-integrated by IPA	44	0.425	2.027	4.25E-04
Rap1	Network-integrated by IPA	50	0.449	1.971	8.78E-04
Rar	Network-integrated by IPA	22	0.389	2.087	3.48E-04
RAS	Network-integrated by IPA	119	0.270	1.798	0.00892141
Ras homolog	Network-integrated by IPA	64	0.361	1.929	0.00182801
Rb	Network-integrated by IPA	36	0.276	2.029	6.80E-04
Rho gdi	Network-integrated by IPA	19	0.382	2.166	2.23E-04
RNA polymerase II	Network-integrated by IPA	46	0.320	1.975	0.00136026
Rnr	Network-integrated by IPA	4	0.500	2.262	3.09E-06
Rock	Network-integrated by IPA	39	0.411	1.998	4.30E-04
RPS6KA	Network-integrated by IPA	21	0.474	2.079	3.80E-04
Rsk	Network-integrated by IPA	30	0.455	2.056	3.70E-04
R-Smad	Network-integrated by IPA	6	0.167	2.782	4.82E-06
Rxr	Network-integrated by IPA	27	0.293	2.118	7.91E-04
S100A1	Network-integrated by IPA	11	0.528	2.249	3.03E-05
SAA	Network-integrated by IPA	31	0.539	2.042	2.39E-04
Sapk	Network-integrated by IPA	21	0.510	2.079	1.21E-04
SCAVENGER receptor CLASS A	Network-integrated by IPA	6	0.800	2.274	7.40E-07
Secretase gamma	Network-integrated by IPA	18	0.483	2.094	6.83E-05
secreted MMP	Network-integrated by IPA	4	0.333	2.694	7.84E-06
Selectin	Network-integrated by IPA	5	0.500	2.478	2.64E-06
Sfk	Network-integrated by IPA	34	0.485	2.010	3.26E-04
SFRP	Network-integrated by IPA	5	0.400	2.809	3.63E-05
Shc	Network-integrated by IPA	46	0.364	1.992	7.46E-04
Smad	Network-integrated by IPA	19	0.419	2.112	3.25E-04
SMAD1/5	Network-integrated by IPA	14	0.484	2.104	1.56E-04
Smad1/5/8	Network-integrated by IPA	12	0.379	2.260	3.86E-04
Smad2/3	Network-integrated by IPA	19	0.419	2.141	9.76E-04
Sod	Network-integrated by IPA	20	0.711	2.060	3.10E-05

somatostatin receptor	Network-integrated by IPA		6	0.333	2.428	1.72E-05
Sos	Network-integrated by IPA		28	0.653	2.048	8.06E-05
sphingomyelinase	Network-integrated by IPA		13	0.769	2.119	1.29E-05
Sphk	Network-integrated by IPA		24	0.525	2.033	1.12E-04
sPla2	Network-integrated by IPA		7	0.667	2.222	1.13E-05
SRC (family)	Network-integrated by IPA		74	0.332	1.898	0.00232033
STAT	Network-integrated by IPA		46	0.318	1.987	9.92E-04
Stat3-Stat3	Network-integrated by IPA		10	0.750	2.216	4.19E-06
STAT5a/b	Network-integrated by IPA		64	0.333	1.933	0.00240339
SYK/ZAP	Network-integrated by IPA		31	0.495	2.042	1.42E-04
TCF	Network-integrated by IPA		12	0.400	2.264	5.27E-05
TCR	Network-integrated by IPA		93	0.258	1.879	0.00448837
TEC/BTK/ITK/TXK/BMX	Network-integrated by IPA		24	0.482	2.087	9.20E-05
Tgf beta	Network-integrated by IPA		101	0.271	1.832	0.00666028
TGFB1	Network-integrated by IPA		230	0.148	1.568	0.05547221
TGFBR	Network-integrated by IPA		20	0.374	2.075	0.00133989
TH1 Cytokine	Network-integrated by IPA		10	0.422	2.239	2.45E-05
TH17 Cytokine	Network-integrated by IPA		6	0.667	2.590	3.59E-06
TH2 Cytokine	Network-integrated by IPA		14	0.363	2.250	6.59E-05
Thrombospondin	Network-integrated by IPA		15	0.400	2.218	1.35E-04
thymidine kinase	Network-integrated by IPA		14	0.143	2.482	1.87E-04
thyroid hormone receptor	Network-integrated by IPA		16	0.319	2.312	8.02E-05
Timp	Network-integrated by IPA		12	0.742	2.123	8.87E-06
Tlr	Network-integrated by IPA		53	0.393	2.000	0.00127370
TLR2/3/4/9	Network-integrated by IPA		7	0.667	2.595	5.65E-06
TLR2/TLR4	Network-integrated by IPA		11	0.636	2.195	1.26E-05
TLR6	Network-integrated by IPA		24	0.693	2.077	3.27E-04
TLR7/8	Network-integrated by IPA		6	0.667	2.187	4.52E-06
Tnf (family)	Network-integrated by IPA		70	0.326	1.913	0.00265730
Tnf receptor	Network-integrated by IPA		29	0.476	2.058	1.79E-04
TRAF	Network-integrated by IPA		11	0.800	2.152	7.63E-06
transglutaminase	Network-integrated by IPA		13	0.667	2.098	4.25E-04
TSH	Network-integrated by IPA		34	0.425	2.027	5.63E-04
Type I BMP receptor	Network-integrated by IPA		5	0.500	2.715	1.81E-05
Type I Receptor	Network-integrated by IPA		4	0.667	2.904	1.84E-05
tyrosine kinase	Network-integrated by IPA		26	0.385	2.087	4.64E-04
Ubiquitin	Network-integrated by IPA		27	0.330	2.094	2.72E-04
VAV	Network-integrated by IPA		38	0.463	2.040	2.75E-04
Vegf	Network-integrated by IPA		133	0.248	1.763	0.01109959
VitaminD3-VDR-RXR	Network-integrated by IPA		6	0.200	2.676	1.60E-05
voltage-gated calcium channel	Network-integrated by IPA		18	0.294	2.297	2.25E-04
Wnt	Network-integrated by IPA		21	0.386	2.135	0.00129485
YAP1	Network-integrated by IPA		44	0.310	1.985	0.00152682
ACVR2A	Downregulated in Secretome	-1.214	22	0.190	2.401	0.00126091
ANGPT2	Downregulated in Secretome	-1.421	59	0.412	1.927	0.00112465
ANGPTL1	Downregulated in Secretome	-1.577	2	1.000	2.453	0
ANGPTL2	Downregulated in Secretome	-1.133	8	0.533	2.241	6.78E-05
BMPR1B	Downregulated in Secretome	-1.172	15	0.295	2.617	3.52E-04

CCL22	Downregulated in Secretome	-1.005	28	0.548	2.044	3.69E-04
CCL27	Downregulated in Secretome	-1.770	9	0.750	2.102	9.35E-06
CCL28	Downregulated in Secretome	-1.460	12	0.394	2.233	2.38E-04
CCL3	Downregulated in Secretome	-2.345	67	0.384	1.919	0.00234097
CCR7	Downregulated in Secretome	-2.313	51	0.400	1.960	0.00199608
CCR9	Downregulated in Secretome	-1.284	15	0.514	2.210	1.56E-04
CHRDL2	Downregulated in Secretome	-1.613	4	0.667	2.900	8.23E-06
CNTF	Downregulated in Secretome	-1.843	33	0.561	2.012	7.33E-04
CXCL11	Downregulated in Secretome	-2.566	39	0.476	2.017	5.40E-04
CXCL14	Downregulated in Secretome	-3.236	16	0.297	2.333	1.92E-04
CXCR2	Downregulated in Secretome	-1.157	36	0.408	2.013	7.03E-04
DLK1	Downregulated in Secretome	-1.638	25	0.372	2.056	0.00105997
EREG	Downregulated in Secretome	-1.114	34	0.343	2.023	0.00212391
FGF23	Downregulated in Secretome	-2.425	11	0.582	2.237	1.86E-05
FGFR4	Downregulated in Secretome	-1.351	25	0.443	2.116	3.56E-04
FGFRL1	Downregulated in Secretome	-2.645	4	0.167	2.318	1.91E-05
FSTL1	Downregulated in Secretome	-2.370	25	0.419	2.031	6.12E-04
GCG	Downregulated in Secretome	-2.026	43	0.300	2.054	0.00150014
GDF15	Downregulated in Secretome	-1.142	32	0.441	2.019	0.00108228
HBEGF	Downregulated in Secretome	-3.074	56	0.357	1.938	0.00448213
IL17C	Downregulated in Secretome	-1.473	13	0.564	2.202	2.96E-05
IL17F	Downregulated in Secretome	-1.571	30	0.402	2.058	4.89E-04
IL18RAP	Downregulated in Secretome	-1.220	21	0.532	2.156	1.75E-04
IL1RN	Downregulated in Secretome	-2.123	69	0.297	1.923	0.00339444
IL21	Downregulated in Secretome	-1.148	66	0.336	1.948	0.00295989
IL23A	Downregulated in Secretome	-1.317	41	0.440	1.994	9.27E-04
IL25	Downregulated in Secretome	-1.288	33	0.385	2.058	0.00164467
LCN1	Downregulated in Secretome	-1.429	1	0	2.726	0
LEPR	Downregulated in Secretome	-1.447	35	0.390	2.027	0.00126805
OSM	Downregulated in Secretome	-1.747	73	0.303	1.894	0.00383233
PTGDR2	Downregulated in Secretome	-1.044	8	0.286	2.503	2.08E-05
PTX3	Downregulated in Secretome	-1.929	23	0.543	2.050	2.64E-04
SIGLEC9	Downregulated in Secretome	-1.436	11	0.417	2.308	2.85E-05
SLC2A1	Downregulated in Secretome	-1.526	36	0.414	1.990	3.23E-04
TGFBR1	Downregulated in Secretome	-1.791	53	0.282	1.952	0.00628753
THBS2	Downregulated in Secretome	-1.723	19	0.426	2.108	4.22E-04
TIMP2	Downregulated in Secretome	-3.051	35	0.434	2.013	0.00169241
TMEFF1	Downregulated in Secretome	-2.251	1	0	2.940	0
TNF	Downregulated in Secretome	-1.314	336	0.105	1.360	0.13084036
TNFRSF11B	Downregulated in Secretome	-1.682	33	0.568	1.985	2.03E-04
TNFSF13	Downregulated in Secretome	-1.822	28	0.298	2.168	0.00146192
TRADD	Downregulated in Secretome	-2.960	29	0.538	2.060	2.00E-04
WFIKKN2	Downregulated in Secretome	-2.062	1	0	2.566	0

Table S2. Enriched secretome network biological processes. The IPA-derived 520 node secretome network was queried through the Cytoscape [3] embedded application BiNGO (**Bi**oinformatic **N**etwork **G**ene **O**ntology) [5] to extract enriched Gene Ontology (GO) biological processes (BP). Specifically, to identify overrepresented BP, a hypergeometric test with Benjamini & Hochberg false discovery rate (B-H FDR) correction with significance cutoff set at $p < 0.001$ and the full GO-BP annotation as background reference. Resulting enriched processes form a nested hierarchy of GO parent and child terms comprising 660 nodes (583 with $p < 0.001$) with 1129 edges. The most significant BP clustered into 8 categories, representing General Processes. Specifically, the top 100 BP are represented by 'Regulation' (56 processes), followed by 'Development' (14), 'Signaling' (8), 'Motility' (8), 'Stimulus/Stress Response' (6), 'Immunity/Inflammation' (5), 'Cellular' (2), and 'Behavior' (1).

Table S2 – Enriched secretome network biological processes

-log (adj p-value)	Rank	BiNGO Biological Process	General process
47.152	1	positive regulation of cellular process	Regulation
46.830	2	positive regulation of biological process	
45.904	3	biological regulation	
44.747	4	regulation of cellular process	
43.320	5	regulation of biological process	
34.980	12	regulation of multicellular organismal process	
31.662	15	regulation of developmental process	
27.254	20	regulation of cell communication	
26.728	21	regulation of cell proliferation	
25.547	25	regulation of signaling pathway	
24.455	27	positive regulation of signal transduction	
24.330	28	positive regulation of signaling process	
24.205	29	positive regulation of cell communication	
24.080	30	negative regulation of biological process	
23.903	31	positive regulation of signaling pathway	
22.977	34	positive regulation of developmental process	
22.028	38	positive regulation of cell proliferation	
21.234	41	negative regulation of cellular process	
19.923	44	regulation of cell differentiation	
18.637	47	positive regulation of immune system process	
18.437	49	regulation of response to stimulus	
17.909	50	positive regulation of cell differentiation	
17.754	51	regulation of phosphorylation	

17.602	52	regulation of protein amino acid phosphorylation	
17.352	53	regulation of phosphorus metabolic process	
17.352	54	regulation of phosphate metabolic process	
17.317	55	regulation of signal transduction	
17.246	56	positive regulation of cellular metabolic process	
17.246	57	positive regulation of intracellular protein kinase cascade	
17.206	58	regulation of signaling process	
17.147	59	positive regulation of metabolic process	
17.045	60	regulation of protein modification process	
16.859	61	regulation of intracellular protein kinase cascade	
16.518	62	regulation of metabolic process	
15.841	66	positive regulation of macromolecule metabolic process	
15.669	67	regulation of MAPKKK cascade	
15.337	68	regulation of biological quality	
15.238	70	positive regulation of cell activation	
14.893	72	regulation of cell activation	
14.882	73	regulation of cellular protein metabolic process	
14.620	74	regulation of lymphocyte activation	
14.545	75	positive regulation of lymphocyte activation	
14.133	79	positive regulation of leukocyte activation	
13.907	80	regulation of cellular metabolic process	
13.888	81	regulation of leukocyte activation	
13.738	82	positive regulation of MAPKKK cascade	
13.485	83	regulation of macromolecule metabolic process	
13.336	84	regulation of protein metabolic process	
13.309	85	positive regulation of molecular function	
13.143	88	positive regulation of multicellular organismal process	
12.846	90	regulation of primary metabolic process	
12.648	91	regulation of growth	
12.368	93	regulation of apoptosis	
12.231	94	regulation of programmed cell death	
12.169	95	regulation of immune response	
12.050	98	regulation of cell death	
42.834	6	developmental process	**Development**
42.436	7	anatomical structure development	
42.436	8	multicellular organismal development	
40.405	9	system development	
34.498	14	organ development	
29.681	17	cellular developmental process	
29.369	19	cell differentiation	
25.686	24	anatomical structure morphogenesis	
18.527	48	tissue development	
14.249	76	nervous system development	
13.195	87	organ morphogenesis	
12.490	92	growth	
11.986	99	blood vessel development	
11.978	100	skeletal system development	
34.511	13	signaling	**Signaling**
25.979	22	signaling pathway	
25.731	23	enzyme linked receptor protein signaling pathway	

25.547	26	cell surface receptor linked signaling pathway	
22.154	36	signaling process	
22.154	37	signal transmission	
18.834	46	signal transduction	
16.518	63	transmembrane receptor protein tyrosine kinase signaling pathway	
22.428	35	locomotion	**Motility**
15.916	65	locomotory behavior	
15.150	71	cellular component movement	
14.249	77	taxis	
14.249	78	chemotaxis	
13.117	89	cell migration	
12.145	96	localization of cell	
12.145	97	cell motility	
35.321	11	response to stimulus	**Stimulus/Stress Response**
21.377	39	response to wounding	
20.428	43	defense response	
19.869	45	response to chemical stimulus	
16.377	64	response to external stimulus	
15.334	69	response to stress	
36.202	10	immune system process	**Immunity/ Inflammation**
29.489	18	immune response	
23.245	33	inflammatory response	
21.321	40	regulation of immune system process	
13.224	86	cell activation	
31.617	16	multicellular organismal process	**Cellular**
23.659	32	cellular process	
20.706	42	behavior	**Behavior**

Table S3. miR-146 dependent network nodes and topological properties. Upstream regulator analysis prioritized miR-146 and a miR-146 dependent regulatory cassette connecting downstream to 101 of the 155 differentially expressed secretome proteins. This 101 protein subset was submitted to IPA with a 2-fold differential expression cutoff for upregulated (orange) or downregulated (blue) proteins. Network-integrated (rose) nodes were derived from the IPA-based network neighborhood, obtained from direct and indirect relationships for derivation of pairwise interactions (edges). Network edges were undirected, unweighted, with multi-edge node pairs merged as a single interaction, resulting in a 430 node network with 6755 edges, with network connectivity demonstrating a power law distribution ($y = 40.08x^{-0.71}$, $r = 0.79$) consistent with non-random scale-free architecture [1,2]. Imported into Cytoscape [3], NetworkAnalyzer [4] was applied to interpret network node topology of the miR-146 dependent subset of the differentially expressed secretome. Listed topological parameters are identical to those listed in Table S1.

Table S3 – miR-146 dependent network nodes and topological properties

Node	Network Inclusion Basis	Log_2 FC	Degree	Clustering Coefficient	Aveerage Shortest Path Length	Betweenness Centrality
ACKR2	Upregulated in Secretome	1.196	12	0.485	2.191	3.19E-04
AGER	Upregulated in Secretome	1.431	42	0.422	1.965	0.00210857
AGRP	Upregulated in Secretome	1.193	5	0.800	2.524	3.41E-05
ALCAM	Upregulated in Secretome	10.46	26	0.257	2.075	0.00126733
ANGPT1	Upregulated in Secretome	1.107	35	0.519	1.995	3.09E-04
AREG	Upregulated in Secretome	1.017	49	0.365	1.963	0.00219055
BIK	Upregulated in Secretome	8.153	10	0.267	2.403	6.55E-05
BPIFA1	Upregulated in Secretome	1.881	5	0.800	2.329	2.67E-06
CCL20	Upregulated in Secretome	1.023	38	0.344	1.991	0.00151276
CCL7	Upregulated in Secretome	1.567	24	0.506	2.049	2.57E-04
CCN3	Upregulated in Secretome	1.011	21	0.327	2.126	6.26E-04
CD14	Upregulated in Secretome	1.404	42	0.394	1.981	9.14E-04
CD163	Upregulated in Secretome	2.891	11	0.250	2.394	2.02E-04
CD40	Upregulated in Secretome	1.213	85	0.255	1.851	0.01201139
CD40LG	Upregulated in Secretome	1.157	83	0.263	1.862	0.00859056
CD80	Upregulated in Secretome	3.153	48	0.392	1.965	0.00336553
CRIM1	Upregulated in Secretome	1.340	14	0.385	2.343	3.53E-04
CSF2RA	Upregulated in Secretome	1.025	12	0.178	2.308	1.87E-04

CTNNB1	Upregulated in Secretome	1.593	127	0.198	1.723	0.02865061
CXCL13	Upregulated in Secretome	1.921	19	0.310	2.119	5.64E-04
CXCL2	Upregulated in Secretome	1.837	51	0.255	1.928	0.00383465
CXCR3	Upregulated in Secretome	1.060	25	0.407	2.082	4.74E-04
DEFB4A/ DEFB4B	Upregulated in Secretome	7.654	22	0.432	2.107	5.74E-04
DKK1	Upregulated in Secretome	2.001	23	0.273	2.110	0.003074
EDAR	Upregulated in Secretome	1.067	3	0.667	2.485	9.90E-07
EGF	Upregulated in Secretome	1.040	148	0.184	1.671	0.0388255
EGFR	Upregulated in Secretome	1.124	138	0.201	1.720	0.02725265
FADD	Upregulated in Secretome	1.010	27	0.410	2.030	0.00115867
FRZB	Upregulated in Secretome	1.019	8	0.200	2.760	3.97E-04
FZD1	Upregulated in Secretome	2.647	11	0.278	2.343	8.43E-05
FZD7	Upregulated in Secretome	1.011	5	0.400	2.590	2.03E-05
HGF	Upregulated in Secretome	1.091	103	0.217	1.779	0.02367151
IGF1R	Upregulated in Secretome	1.249	90	0.263	1.825	0.01210658
IGFBP3	Upregulated in Secretome	1.050	48	0.320	1.974	0.00423877
IGFBP6	Upregulated in Secretome	1.134	11	0.218	2.261	2.84E-04
IGFBP7	Upregulated in Secretome	1.701	12	0.409	2.207	2.06E-04
IL17RA	Upregulated in Secretome	1.134	24	0.319	2.135	0.00266359
IL17RB	Upregulated in Secretome	2.584	9	0.500	2.357	5.96E-04
IL17RD	Upregulated in Secretome	1.071	6	0.933	2.319	1.40E-07
IL18R1	Upregulated in Secretome	2.067	25	0.470	2.070	4.65E-04
IL1B	Upregulated in Secretome	1.095	190	0.151	1.573	0.07097365
IL1R2	Upregulated in Secretome	1.121	19	0.275	2.142	0.00104414
IL21R	Upregulated in Secretome	1.708	18	0.350	2.438	1.88E-04
IL2RA	Upregulated in Secretome	2.572	55	0.414	1.918	0.00174296
IL5RA	Upregulated in Secretome	1.208	14	0.364	2.375	1.79E-04
IL7R	Upregulated in Secretome	1.805	32	0.369	2.028	8.95E-04
IL9	Upregulated in Secretome	1.777	42	0.295	2.002	0.00243335
ITGAL	Upregulated in Secretome	1.051	27	0.417	2.049	9.88E-04
LEP	Upregulated in Secretome	2.369	87	0.239	1.858	0.01260958
MMP9	Upregulated in Secretome	1.346	133	0.214	1.711	0.02980729
NGF	Upregulated in Secretome	1.427	73	0.236	1.865	0.00684951
PDGFA	Upregulated in Secretome	1.971	24	0.338	2.124	5.24E-04
PF4	Upregulated in Secretome	2.252	30	0.423	2.075	7.01E-04
PRL	Upregulated in Secretome	1.093	78	0.312	1.851	0.00931091
SELE	Upregulated in Secretome	1.797	57	0.298	1.897	0.00421343
SELP	Upregulated in Secretome	1.292	30	0.362	2.086	5.61E-04
SFRP4	Upregulated in Secretome	1.480	7	0.429	2.541	0.00116709
SMAD1	Upregulated in Secretome	3.058	36	0.244	2.030	0.00560806
SMAD9	Upregulated in Secretome	2.324	6	0.333	2.918	2.40E-05
TFPI	Upregulated in Secretome	1.073	3	0	2.657	8.89E-06
TLR3	Upregulated in Secretome	1.276	12	0.273	2.333	3.38E-04
TLR4	Upregulated in Secretome	1.350	26	0.400	2.098	9.98E-04
TNFAIP6	Upregulated in Secretome	1.049	14	0.451	2.124	3.08E-04
TNFRSF10A	Upregulated in Secretome	1.719	10	0.444	2.198	1.40E-04
TNFRSF6B	Upregulated in Secretome	1.005	1	0	2.776	0
TNFSF11	Upregulated in Secretome	1.724	33	0.420	1.991	6.63E-04
TYMP	Upregulated in Secretome	1.452	6	0.600	2.336	4.54E-06
26s Proteasome	Network-integrated by IPA		47	0.283	1.972	0.00330715

7S NGF	Network-integrated by IPA	5	0.300	2.434	2.75E-05
Actin	Network-integrated by IPA	51	0.281	1.949	0.0023036
ADCY	Network-integrated by IPA	37	0.386	1.967	0.0015548
adhesion molecule	Network-integrated by IPA	11	0.709	2.168	7.83E-05
ADRB	Network-integrated by IPA	24	0.398	2.079	5.78E-04
Akt	Network-integrated by IPA	199	0.165	1.548	0.05522428
ALDH	Network-integrated by IPA	2	0	2.688	1.64E-06
Alp	Network-integrated by IPA	30	0.446	1.991	8.64E-04
Alpha Actinin	Network-integrated by IPA	17	0.400	2.226	7.65E-05
Alpha catenin	Network-integrated by IPA	33	0.312	2.012	0.00118547
ALT	Network-integrated by IPA	6	0.133	2.541	3.48E-05
Ampa Receptor	Network-integrated by IPA	20	0.451	2.156	9.64E-05
AMPK	Network-integrated by IPA	52	0.336	1.963	0.00192978
amylase	Network-integrated by IPA	4	0.333	2.508	4.68E-06
Angiotensin II receptor type 1	Network-integrated by IPA	16	0.495	2.133	7.11E-05
Ap1	Network-integrated by IPA	102	0.295	1.779	0.01110244
Ap2	Network-integrated by IPA	6	0.800	2.273	2.07E-06
Atrial Natriuretic Peptide	Network-integrated by IPA	8	0.607	2.249	2.14E-05
ATXN7	Network-integrated by IPA	4	0.500	2.718	3.54E-05
BCR (complex)	Network-integrated by IPA	69	0.268	1.914	0.00515789
C/EBP	Network-integrated by IPA	17	0.629	2.124	5.66E-05
C1q	Network-integrated by IPA	22	0.516	2.124	2.15E-04
C8	Network-integrated by IPA	4	0.500	2.492	5.61E-06
CACNA1A	Network-integrated by IPA	10	0.607	2.226	5.30E-05
CALC	Network-integrated by IPA	15	0.667	2.121	6.76E-05
Calcineurin A	Network-integrated by IPA	13	0.618	2.145	5.75E-05
Calcineurin protein(s)	Network-integrated by IPA	42	0.344	1.979	0.00141179
Calmodulin	Network-integrated by IPA	49	0.361	1.942	0.00363128
calpain	Network-integrated by IPA	39	0.312	2.002	0.00149099
CaMKII	Network-integrated by IPA	40	0.424	1.986	7.82E-04
Casein	Network-integrated by IPA	9	0.571	2.247	1.96E-05
caspase	Network-integrated by IPA	51	0.348	1.923	0.00200496
Caspase 3/7	Network-integrated by IPA	8	0.286	2.434	3.12E-05
Cbp/p300	Network-integrated by IPA	23	0.332	2.105	8.47E-04
C-C chemokine receptor	Network-integrated by IPA	9	0.429	2.571	2.34E-05
CD3	Network-integrated by IPA	84	0.250	1.851	0.00856483
CD3 group	Network-integrated by IPA	17	0.522	2.114	6.28E-05
CD4	Network-integrated by IPA	41	0.298	1.998	0.00171215
CD80/CD86	Network-integrated by IPA	2	1.000	2.802	0
CD9	Network-integrated by IPA	27	0.450	2.058	2.66E-04
Cebp	Network-integrated by IPA	4	0.833	2.326	3.17E-06
CG	Network-integrated by IPA	42	0.404	1.963	0.00191417
chemokine	Network-integrated by IPA	40	0.282	2.044	0.00215174
chemokine receptor	Network-integrated by IPA	10	0.357	2.448	3.21E-05
Ciap	Network-integrated by IPA	12	0.545	2.154	4.13E-05
Ck2	Network-integrated by IPA	27	0.227	2.110	0.00134361
Cofilin	Network-integrated by IPA	28	0.431	2.040	4.12E-04
collagen	Network-integrated by IPA	18	0.366	2.135	3.03E-04

Collagen Alpha1	Network-integrated by IPA	21	0.503	2.093	2.99E-04
Collagen type I (complex)	Network-integrated by IPA	38	0.476	1.979	0.00107657
collagen type I (family)	Network-integrated by IPA	23	0.336	2.054	9.41E-04
Collagen type II	Network-integrated by IPA	14	0.516	2.175	2.13E-04
Collagen type III	Network-integrated by IPA	7	0.714	2.333	1.10E-05
Collagen type IV	Network-integrated by IPA	24	0.290	2.193	4.66E-04
Collagen(s)	Network-integrated by IPA	45	0.338	1.963	0.00177709
Complement	Network-integrated by IPA	13	0.455	2.319	4.49E-05
Cpla2	Network-integrated by IPA	21	0.543	2.049	9.75E-05
CPT1	Network-integrated by IPA	8	0.267	2.723	2.58E-05
Cr3	Network-integrated by IPA	19	0.456	2.154	1.10E-04
Creb	Network-integrated by IPA	85	0.323	1.825	0.00560351
CREB1	Network-integrated by IPA	73	0.313	1.865	0.00782967
CREBBP	Network-integrated by IPA	72	0.331	1.855	0.00519244
c-Src	Network-integrated by IPA	11	0.556	2.207	1.19E-05
Cyclin A	Network-integrated by IPA	34	0.401	1.979	5.75E-04
Cyclin D	Network-integrated by IPA	25	0.514	2.124	4.87E-04
Cyclin E	Network-integrated by IPA	24	0.511	2.044	2.16E-04
cyclooxygenase	Network-integrated by IPA	14	0.560	2.100	1.92E-04
CYP19	Network-integrated by IPA	10	0.714	2.170	9.68E-06
cytochrome C	Network-integrated by IPA	27	0.397	2.082	4.69E-04
cytokine	Network-integrated by IPA	121	0.199	1.748	0.01927696
cytokine receptor	Network-integrated by IPA	20	0.176	2.357	4.20E-04
DNA-PK	Network-integrated by IPA	8	0.600	2.287	9.16E-06
Dynamin	Network-integrated by IPA	12	0.697	2.186	3.05E-05
E2f	Network-integrated by IPA	18	0.467	2.093	2.26E-04
Ecm	Network-integrated by IPA	9	0.333	2.436	3.52E-05
EGFR ligand	Network-integrated by IPA	9	0.611	2.415	2.80E-05
EGFR/PDGFR/IGFR	Network-integrated by IPA	7	0.810	2.347	8.87E-06
EGLN	Network-integrated by IPA	6	0.200	2.578	9.08E-05
elastase	Network-integrated by IPA	15	0.505	2.156	2.94E-04
EOMES	Network-integrated by IPA	19	0.294	2.186	9.99E-04
Eotaxin	Network-integrated by IPA	4	0.833	2.401	2.71E-06
EP300	Network-integrated by IPA	82	0.226	1.834	0.01637025
ERBB	Network-integrated by IPA	12	0.455	2.319	4.35E-05
Erbb1 dimer	Network-integrated by IPA	5	0.600	2.541	5.56E-06
Erbb1-Erbb4 dimer	Network-integrated by IPA	2	1.000	2.890	0
ERK	Network-integrated by IPA	142	0.202	1.702	0.02154021
ERK1/2	Network-integrated by IPA	169	0.176	1.615	0.03875768
Erm	Network-integrated by IPA	16	0.538	2.191	4.09E-05
ESR1	Network-integrated by IPA	112	0.232	1.760	0.01689657
ESR2	Network-integrated by IPA	63	0.286	1.895	0.00730406
estrogen receptor	Network-integrated by IPA	53	0.329	1.937	0.00303107
ETS	Network-integrated by IPA	11	0.655	2.133	6.75E-05
F Actin	Network-integrated by IPA	40	0.317	2.012	9.29E-04
farnesyl transferase	Network-integrated by IPA	7	0.619	2.438	6.86E-06
Fascin	Network-integrated by IPA	12	0.644	2.152	1.72E-05
Fc gamma receptor	Network-integrated by IPA	32	0.401	2.016	7.78E-04
Fcer1	Network-integrated by IPA	38	0.400	1.995	0.00150239

Fcgr2	Network-integrated by IPA	9	0.639	2.256	1.52E-05
Fcgr3	Network-integrated by IPA	11	0.436	2.266	4.72E-05
Fgf	Network-integrated by IPA	21	0.474	2.128	3.02E-04
Fibrin	Network-integrated by IPA	9	0.444	2.480	3.10E-05
Fibrinogen	Network-integrated by IPA	23	0.368	2.075	5.74E-04
FKHR	Network-integrated by IPA	6	0.733	2.261	4.10E-06
Focal adhesion kinase	Network-integrated by IPA	57	0.345	1.918	0.00286413
Foxo	Network-integrated by IPA	10	0.643	2.266	2.12E-05
Frizzled	Network-integrated by IPA	14	0.273	2.361	0.00247195
FSH	Network-integrated by IPA	47	0.383	1.935	0.00227947
G protein	Network-integrated by IPA	46	0.257	1.995	0.00359183
G protein alphai	Network-integrated by IPA	31	0.485	2.033	4.44E-04
G protein beta gamma	Network-integrated by IPA	25	0.570	2.079	2.37E-04
Gap	Network-integrated by IPA	13	0.800	2.207	1.21E-05
GC-GCR dimer	Network-integrated by IPA	10	0.267	2.324	2.02E-04
gelatinase	Network-integrated by IPA	8	0.679	2.266	1.25E-05
Glycogen synthase	Network-integrated by IPA	17	0.514	2.245	1.14E-04
GNRH	Network-integrated by IPA	14	0.549	2.152	4.87E-05
GOT	Network-integrated by IPA	6	0.800	2.333	2.37E-06
Gpcr	Network-integrated by IPA	51	0.357	1.974	0.00296875
G-protein beta	Network-integrated by IPA	23	0.486	2.172	1.91E-04
growth factor	Network-integrated by IPA	29	0.537	2.033	3.16E-04
growth factor receptor	Network-integrated by IPA	21	0.520	2.205	1.67E-04
Growth hormone	Network-integrated by IPA	48	0.289	1.984	0.00340227
Gsk3	Network-integrated by IPA	58	0.370	1.900	0.00296128
Hdac	Network-integrated by IPA	33	0.262	2.028	0.00174084
HDL	Network-integrated by IPA	27	0.427	2.044	6.00E-04
hemoglobin	Network-integrated by IPA	12	0.222	2.259	0.00174211
Hif1	Network-integrated by IPA	24	0.329	2.112	2.93E-04
HISTONE	Network-integrated by IPA	20	0.359	2.098	2.66E-04
histone deacetylase	Network-integrated by IPA	13	0.154	2.366	4.68E-04
Histone h3	Network-integrated by IPA	70	0.337	1.862	0.00394624
Histone h4	Network-integrated by IPA	42	0.313	1.953	0.00189755
HLA-DR	Network-integrated by IPA	19	0.585	2.166	9.18E-05
HLA-DR group	Network-integrated by IPA	8	0.607	2.301	1.96E-05
HRAS	Network-integrated by IPA	100	0.282	1.802	0.0092741
Hsp27	Network-integrated by IPA	43	0.421	1.965	0.00128234
Hsp70	Network-integrated by IPA	48	0.371	1.930	0.00162977
Hsp90	Network-integrated by IPA	55	0.369	1.911	0.00205018
Hspg	Network-integrated by IPA	7	0.286	2.417	3.21E-05
I kappa b kinase	Network-integrated by IPA	16	0.558	2.082	2.51E-04
Icam	Network-integrated by IPA	4	0.333	2.541	3.16E-05
Ifn	Network-integrated by IPA	33	0.286	2.044	0.00126546
IFN alpha/beta	Network-integrated by IPA	19	0.456	2.138	3.87E-04
IFN Beta	Network-integrated by IPA	55	0.342	1.932	0.00308099
Ifn gamma	Network-integrated by IPA	28	0.365	2.070	5.51E-04
IFN type 1	Network-integrated by IPA	19	0.357	2.203	5.14E-04
Ifnar	Network-integrated by IPA	16	0.458	2.254	2.41E-04
Iga	Network-integrated by IPA	28	0.458	2.138	9.20E-04

IgD	Network-integrated by IPA	9	0.810	2.508	2.02E-06
Ige	Network-integrated by IPA	70	0.290	1.893	0.00545687
Igf	Network-integrated by IPA	13	0.603	2.193	2.69E-04
Igfbp	Network-integrated by IPA	8	0.250	2.571	6.54E-05
IgG	Network-integrated by IPA	73	0.280	1.862	0.00650834
IgG1	Network-integrated by IPA	27	0.453	2.086	5.53E-04
IgG2a	Network-integrated by IPA	23	0.455	2.110	5.96E-04
IgG2b	Network-integrated by IPA	12	0.591	2.312	8.10E-05
Igg3	Network-integrated by IPA	13	0.410	2.354	1.05E-04
Igm	Network-integrated by IPA	47	0.383	1.984	0.00193656
Ikb	Network-integrated by IPA	31	0.414	2.014	6.50E-04
IKK (complex)	Network-integrated by IPA	39	0.445	1.944	7.21E-04
Ikk (family)	Network-integrated by IPA	21	0.532	2.091	2.69E-04
IL1	Network-integrated by IPA	99	0.242	1.809	0.01784513
IL12 (complex)	Network-integrated by IPA	74	0.304	1.881	0.00678608
IL12 (family)	Network-integrated by IPA	59	0.311	1.935	0.00272977
IL12RB2/IL23R	Network-integrated by IPA	4	1.000	2.459	0
IL17a dimer	Network-integrated by IPA	9	0.556	2.431	2.83E-05
IL-17f dimer	Network-integrated by IPA	6	0.333	2.506	2.00E-05
IL17R	Network-integrated by IPA	15	0.343	2.196	4.70E-04
IL-1R	Network-integrated by IPA	24	0.511	2.075	2.08E-04
IL-1R/TLR	Network-integrated by IPA	4	0.333	2.741	1.24E-05
IL23	Network-integrated by IPA	26	0.412	2.128	0.00111618
Il8r	Network-integrated by IPA	14	0.473	2.131	1.05E-04
Immunoglobulin	Network-integrated by IPA	108	0.225	1.779	0.01393124
Importin alpha	Network-integrated by IPA	9	0.417	2.469	3.26E-05
Insulin	Network-integrated by IPA	113	0.217	1.758	0.01893485
Integrin	Network-integrated by IPA	55	0.338	1.988	0.00295009
Integrin alpha V beta 3	Network-integrated by IPA	12	0.288	2.350	8.27E-05
Interferon alpha	Network-integrated by IPA	70	0.309	1.895	0.00517109
INTERLEUKIN	Network-integrated by IPA	3	0.667	3.012	1.56E-06
IRAK	Network-integrated by IPA	15	0.449	2.198	1.48E-04
IRF	Network-integrated by IPA	14	0.385	2.270	1.91E-04
ITK	Network-integrated by IPA	35	0.330	2.049	0.00189917
JAK	Network-integrated by IPA	36	0.307	2.023	0.00195633
JAK1/2	Network-integrated by IPA	7	0.238	2.361	8.75E-05
JINK1/2	Network-integrated by IPA	20	0.392	2.175	2.90E-04
Jnk	Network-integrated by IPA	121	0.216	1.755	0.0165264
L1CAM	Network-integrated by IPA	24	0.398	2.040	5.68E-04
Laminin (complex)	Network-integrated by IPA	32	0.407	2.042	8.27E-04
Laminin1	Network-integrated by IPA	12	0.530	2.196	9.62E-05
Ldh (complex)	Network-integrated by IPA	19	0.351	2.112	2.91E-04
LDL	Network-integrated by IPA	63	0.355	1.904	0.00284668
Lfa-1	Network-integrated by IPA	11	0.278	2.434	3.66E-05
Lh	Network-integrated by IPA	41	0.472	2.021	0.00135084
Lymphotoxin	Network-integrated by IPA	8	0.393	2.284	1.37E-04
MAC	Network-integrated by IPA	17	0.449	2.091	5.49E-04
MAFB	Network-integrated by IPA	12	0.356	2.275	1.19E-04
MAP2K1	Network-integrated by IPA	79	0.348	1.841	0.00467466
MAP2K1/2	Network-integrated by IPA	50	0.384	1.946	0.00168151

Mapk	Network-integrated by IPA	132	0.223	1.706	0.0194238
MEF2	Network-integrated by IPA	15	0.305	2.184	1.59E-04
Mek	Network-integrated by IPA	57	0.426	1.918	0.00185724
Metalloprotease	Network-integrated by IPA	22	0.377	2.098	5.86E-04
MHC Class I (complex)	Network-integrated by IPA	12	0.364	2.336	1.04E-04
MHC CLASS I (family)	Network-integrated by IPA	15	0.269	2.282	1.75E-04
MHC Class II (complex)	Network-integrated by IPA	29	0.410	2.124	4.11E-04
MHC II	Network-integrated by IPA	12	0.500	2.212	1.55E-04
mir-148	Network-integrated by IPA	8	0.214	2.389	1.37E-04
miR-17-5p (and other miRNAs w/seed AAAGUGC)	Network-integrated by IPA	15	0.267	2.252	3.75E-04
mir-192	Network-integrated by IPA	6	0.333	2.399	2.91E-05
mir-214	Network-integrated by IPA	7	0.238	2.527	5.05E-05
MKK3/6	Network-integrated by IPA	9	0.583	2.270	2.11E-05
Mlc	Network-integrated by IPA	31	0.382	2.044	4.47E-04
Mmp	Network-integrated by IPA	57	0.328	1.900	0.00393976
MTORC1	Network-integrated by IPA	34	0.333	2.023	0.00245456
Mucin	Network-integrated by IPA	11	0.236	2.242	7.98E-05
MYCN	Network-integrated by IPA	29	0.299	2.082	0.00116994
myosin-light-chain kinase	Network-integrated by IPA	16	0.505	2.263	5.58E-05
NADPH oxidase	Network-integrated by IPA	27	0.623	2.047	1.25E-04
N-Cadherin	Network-integrated by IPA	8	0.857	2.172	5.17E-06
N-cor	Network-integrated by IPA	17	0.287	2.228	4.98E-04
NFAT (complex)	Network-integrated by IPA	22	0.468	2.103	1.53E-04
Nfat (family)	Network-integrated by IPA	50	0.357	1.967	0.00145014
NFkB (complex)	Network-integrated by IPA	201	0.163	1.543	0.06071512
NFkB (family)	Network-integrated by IPA	23	0.505	2.005	3.49E-04
NfkB1-RelA	Network-integrated by IPA	17	0.429	2.154	1.45E-04
NfkB-RelA	Network-integrated by IPA	10	0.607	2.322	2.05E-05
Ngf	Network-integrated by IPA	15	0.436	2.240	1.50E-04
Nos	Network-integrated by IPA	35	0.409	1.993	0.00120638
Notch	Network-integrated by IPA	28	0.406	2.033	4.06E-04
Nr1h	Network-integrated by IPA	17	0.267	2.200	6.91E-04
P glycoprotein	Network-integrated by IPA	22	0.463	2.075	1.96E-04
P38 MAPK	Network-integrated by IPA	180	0.180	1.590	0.04487984
p70 S6k	Network-integrated by IPA	39	0.486	1.960	6.73E-04
p85 (pik3r)	Network-integrated by IPA	43	0.482	1.953	4.18E-04
Pak	Network-integrated by IPA	24	0.532	2.100	1.94E-04
PARP	Network-integrated by IPA	18	0.353	2.184	2.95E-04
Pde4	Network-integrated by IPA	11	0.600	2.182	8.02E-05
Pdgf (complex)	Network-integrated by IPA	59	0.300	1.960	0.00362583
PDGF (family)	Network-integrated by IPA	8	0.464	2.277	2.55E-05
Pdgf Ab	Network-integrated by IPA	17	0.463	2.149	1.20E-04
PDGF BB	Network-integrated by IPA	56	0.327	1.967	0.00439077
PDGF-AA	Network-integrated by IPA	11	0.691	2.184	2.48E-05
Pdgfr	Network-integrated by IPA	38	0.446	2.058	5.68E-04
PEPCK	Network-integrated by IPA	18	0.268	2.312	3.35E-04
peptidase	Network-integrated by IPA	3	0.667	2.382	3.30E-07

PI3K (complex)	Network-integrated by IPA	174	0.194	1.611	0.0369184
PI3K (family)	Network-integrated by IPA	44	0.410	1.942	9.67E-04
PI3K beta	Network-integrated by IPA	14	0.484	2.168	1.68E-04
PI3K p85	Network-integrated by IPA	28	0.406	2.075	4.66E-04
Pka	Network-integrated by IPA	102	0.231	1.790	0.01403193
Pka catalytic subunit	Network-integrated by IPA	19	0.434	2.152	2.01E-04
Pkc(s)	Network-integrated by IPA	132	0.224	1.706	0.0216697
PLA2	Network-integrated by IPA	15	0.533	2.198	7.65E-05
PLC	Network-integrated by IPA	45	0.305	1.942	0.00191488
PLC gamma	Network-integrated by IPA	42	0.355	1.986	0.00205206
Pld	Network-integrated by IPA	43	0.370	1.972	0.00119474
potassium channel	Network-integrated by IPA	6	0.667	2.315	3.33E-06
PP1 protein complex group	Network-integrated by IPA	32	0.437	2.054	5.13E-04
PP2A	Network-integrated by IPA	47	0.399	1.958	0.00111421
Ppp2c	Network-integrated by IPA	16	0.615	2.152	4.23E-05
PRKAA	Network-integrated by IPA	18	0.510	2.154	4.61E-04
Pro-inflammatory Cytokine	Network-integrated by IPA	31	0.432	2.016	8.03E-04
Proinsulin	Network-integrated by IPA	20	0.368	2.077	0.00340287
PROTEASE	Network-integrated by IPA	7	0.667	2.294	6.62E-06
protein phosphatase	Network-integrated by IPA	9	0.556	2.336	2.91E-05
PTK	Network-integrated by IPA	23	0.433	2.089	2.35E-04
Rab5	Network-integrated by IPA	9	0.583	2.249	2.29E-05
Rac	Network-integrated by IPA	68	0.309	1.890	0.00422844
Raf	Network-integrated by IPA	39	0.443	2.028	5.80E-04
Rap1	Network-integrated by IPA	37	0.518	2.026	4.24E-04
RAS	Network-integrated by IPA	104	0.279	1.781	0.01098207
Ras homolog	Network-integrated by IPA	53	0.374	1.935	0.00201869
RNA polymerase II	Network-integrated by IPA	44	0.339	1.946	0.00190489
Rock	Network-integrated by IPA	31	0.374	2.030	4.29E-04
RPS6KA	Network-integrated by IPA	16	0.440	2.193	2.33E-04
Rsk	Network-integrated by IPA	30	0.478	2.014	3.07E-04
R-Smad	Network-integrated by IPA	6	0.167	2.762	9.22E-06
RUNX3	Network-integrated by IPA	32	0.232	2.037	0.0028094
Rxr	Network-integrated by IPA	18	0.375	2.226	2.80E-04
S100A1	Network-integrated by IPA	13	0.473	2.235	1.08E-04
S100B	Network-integrated by IPA	22	0.532	2.079	1.97E-04
SAA	Network-integrated by IPA	20	0.529	2.091	8.30E-04
Sapk	Network-integrated by IPA	16	0.542	2.152	7.26E-05
SCAVENGER receptor CLASS A	Network-integrated by IPA	5	0.700	2.473	1.20E-06
Secretase gamma	Network-integrated by IPA	15	0.436	2.172	6.86E-05
secreted MMP	Network-integrated by IPA	1	0	2.709	0
Sfk	Network-integrated by IPA	30	0.467	2.000	4.30E-04
SFRP	Network-integrated by IPA	4	0.500	3.196	2.45E-05
Shc	Network-integrated by IPA	42	0.385	1.991	0.00105954
Smad	Network-integrated by IPA	17	0.352	2.291	0.00100901
SMAD1/5	Network-integrated by IPA	9	0.278	2.312	1.22E-04
Smad1/5/8	Network-integrated by IPA	9	0.472	2.361	4.90E-04
Smad2/3	Network-integrated by IPA	14	0.303	2.186	1.76E-04

SMARCD3	Network-integrated by IPA		11	0.444	2.289	6.23E-05
SMO	Network-integrated by IPA		15	0.282	2.280	3.36E-04
Sod	Network-integrated by IPA		15	0.543	2.140	1.06E-04
somatostatin receptor	Network-integrated by IPA		5	0.200	2.695	1.44E-05
Sos	Network-integrated by IPA		22	0.641	2.152	6.18E-05
sphingomyelinase	Network-integrated by IPA		13	0.628	2.096	3.08E-05
Sphk	Network-integrated by IPA		21	0.410	2.075	3.47E-04
sPla2	Network-integrated by IPA		4	0.667	2.359	7.46E-06
SRC (family)	Network-integrated by IPA		71	0.277	1.879	0.00593865
STAT	Network-integrated by IPA		37	0.341	1.998	0.00108673
Stat3-Stat3	Network-integrated by IPA		10	0.679	2.214	1.06E-05
STAT5a/b	Network-integrated by IPA		58	0.295	1.904	0.00442562
SYK/ZAP	Network-integrated by IPA		26	0.460	2.077	2.12E-04
TCR	Network-integrated by IPA		65	0.256	1.911	0.00465514
TEC/BTK/ITK/TXK/BMX	Network-integrated by IPA		7	0.381	2.455	1.85E-05
Tgf beta	Network-integrated by IPA		17	0.478	2.103	1.59E-04
TGFBR	Network-integrated by IPA		3	0.333	2.580	1.22E-05
TH1 Cytokine	Network-integrated by IPA		4	0.667	2.471	3.59E-06
TH17 Cytokine	Network-integrated by IPA		3	0.333	2.727	4.98E-06
TH2 Cytokine	Network-integrated by IPA		5	0.400	2.378	3.77E-05
thymidine kinase	Network-integrated by IPA		11	0.382	2.403	1.25E-04
Timp	Network-integrated by IPA		4	0.333	2.501	2.85E-06
Tlr	Network-integrated by IPA		4	0.833	2.683	2.57E-06
Tnf (family)	Network-integrated by IPA		17	0.404	2.186	1.84E-04
Tnf receptor	Network-integrated by IPA		2	0	2.704	4.70E-07
TRAF	Network-integrated by IPA		3	1.000	2.462	0
transglutaminase	Network-integrated by IPA		6	0.933	2.387	5.70E-07
TRIM25	Network-integrated by IPA		4	0.500	2.448	2.57E-06
TSH	Network-integrated by IPA		5	0.500	2.597	2.84E-05
tyrosine kinase	Network-integrated by IPA		8	0.179	2.378	4.47E-05
Ubiquitin	Network-integrated by IPA		6	0	2.457	1.75E-04
VAV	Network-integrated by IPA		19	0.345	2.184	1.73E-04
Vegf	Network-integrated by IPA		89	0.284	1.807	0.00851989
VEGFA	Network-integrated by IPA		78	0.343	1.825	0.00681207
VitaminD3-VDR-RXR	Network-integrated by IPA		1	0	2.832	0
voltage-gated calcium channel	Network-integrated by IPA		13	0.359	2.287	2.55E-04
Wnt	Network-integrated by IPA		10	0.244	2.503	0.00144739
ACVR2A	Downregulated in Secretome	-1.214	12	0.212	2.392	2.51E-04
ANGPT2	Downregulated in Secretome	-1.421	58	0.355	1.902	0.00303873
ANGPTL2	Downregulated in Secretome	-1.133	9	0.619	2.270	2.69E-05
CCL22	Downregulated in Secretome	-1.005	23	0.644	2.068	1.75E-04
CCL27	Downregulated in Secretome	-1.770	6	0.800	2.198	5.87E-06
CCL28	Downregulated in Secretome	-1.460	9	0.361	2.469	1.94E-04
CCL3	Downregulated in Secretome	-2.345	57	0.365	1.925	0.00352633
CCR7	Downregulated in Secretome	-2.313	40	0.399	1.970	0.00193678
CNTF	Downregulated in Secretome	-1.843	28	0.437	2.056	0.00102244
CXCL11	Downregulated in Secretome	-2.566	28	0.526	2.051	5.38E-04
CXCL14	Downregulated in Secretome	-3.236	12	0.311	2.366	2.01E-04
CXCR2	Downregulated in Secretome	-1.157	28	0.342	2.084	8.51E-04

DLK1	Downregulated in Secretome	-1.638	18	0.408	2.149	1.94E-04
EREG	Downregulated in Secretome	-1.114	23	0.338	2.112	0.0018301
FGFR4	Downregulated in Secretome	-1.351	19	0.493	2.142	2.03E-04
FGFRL1	Downregulated in Secretome	-2.645	2	0	3.019	5.76E-06
GDF15	Downregulated in Secretome	-1.142	26	0.391	2.093	0.00114602
HBEGF	Downregulated in Secretome	-3.074	52	0.343	1.928	0.00694583
IL17C	Downregulated in Secretome	-1.473	11	0.556	2.308	2.20E-05
IL17F	Downregulated in Secretome	-1.571	26	0.333	2.086	7.08E-04
IL1RN	Downregulated in Secretome	-2.123	41	0.266	2.026	0.00361853
IL21	Downregulated in Secretome	-1.148	62	0.288	1.935	0.00479293
IL23A	Downregulated in Secretome	-1.317	36	0.394	1.974	9.62E-04
IL25	Downregulated in Secretome	-1.288	28	0.397	2.112	0.00291219
LEPR	Downregulated in Secretome	-1.447	31	0.335	2.091	0.00173223
OSM	Downregulated in Secretome	-1.747	69	0.254	1.883	0.00853502
PTX3	Downregulated in Secretome	-1.929	19	0.559	2.117	1.22E-04
SLC2A1	Downregulated in Secretome	-1.526	24	0.420	2.077	3.40E-04
TGFBR1	Downregulated in Secretome	-1.791	18	0.320	2.205	3.78E-04
THBS2	Downregulated in Secretome	-1.723	8	0.357	2.340	1.69E-05
TIMP2	Downregulated in Secretome	-3.051	17	0.375	2.156	1.94E-04
TNF	Downregulated in Secretome	-1.314	139	0.190	1.690	0.02829163
TNFRSF11B	Downregulated in Secretome	-1.682	15	0.600	2.149	3.60E-05
TRADD	Downregulated in Secretome	-2.960	9	0.611	2.235	1.53E-05

Table S4. Enriched miR-146 dependent network biological processes. In Cytoscape [3], BiNGO [5] was used to interpret enriched GO-BP of the IPA-derived 430 node miR-146 dependent network. Using a hypergeometric test with B-H FDR correction and significance cutoff at $p < 0.001$ with the full GO-BP annotation as background reference, the secretome network was surveyed for overrepresented BP. The resulting BiNGO network comprised 619 nodes (558 with $p < 0.001$) with 1077 edges connecting a GO parent and child term nested hierarchy. As in the full secretome BiNGO analysis, the 100 most significant BP of the miR-146 dependent network clustered into 8 general process categories, which are listed by categories appearing most frequently and BP within each category listed by -log *p*-value in descending order of significance. Specifically, the top 100 BP are represented by 'Regulation' (61 processes), followed by 'Development' (18), 'Signaling' (8), 'Stimulus/Stress Response' (5), 'Immunity/Inflammation' (4), 'Cellular' (2), 'Motility' (1), and 'Behavior' (1).

Table S4 – Enriched miR-146 dependent network biological processes

-log (adj p-value)	Rank	BiNGO Biological Process	General process
52.513	1	positive regulation of biological process	
52.513	2	positive regulation of cellular process	
40.051	5	biological regulation	
39.383	6	regulation of cellular process	
38.560	9	regulation of biological process	
34.979	12	regulation of multicellular organismal process	
29.375	19	regulation of cell communication	
29.043	20	regulation of developmental process	
28.012	21	regulation of cell proliferation	
26.620	23	negative regulation of biological process	
26.533	24	positive regulation of metabolic process	**Regulation**
25.976	25	regulation of metabolic process	
25.498	26	positive regulation of cellular metabolic process	
24.851	28	positive regulation of macromolecule metabolic process	
23.935	29	negative regulation of cellular process	
23.230	30	positive regulation of cell communication	
23.096	31	regulation of cellular metabolic process	
22.886	32	positive regulation of cell proliferation	
22.592	34	positive regulation of signal transduction	
22.543	35	positive regulation of developmental process	
22.493	36	positive regulation of signaling process	

22.348	37	regulation of macromolecule metabolic process	
22.127	38	regulation of signaling pathway	
21.116	40	positive regulation of signaling pathway	
20.719	41	regulation of primary metabolic process	
18.413	46	regulation of protein amino acid phosphorylation	
18.302	47	regulation of signal transduction	
18.266	48	regulation of phosphorylation	
18.192	49	regulation of signaling process	
18.008	51	positive regulation of biosynthetic process	
17.909	52	regulation of phosphate metabolic process	
17.909	53	regulation of phosphorus metabolic process	
17.888	54	regulation of cell death	
17.888	55	regulation of cell differentiation	
17.839	57	positive regulation of macromolecule biosynthetic process	
17.224	59	regulation of apoptosis	
17.190	60	positive regulation of cellular biosynthetic process	
17.100	64	regulation of programmed cell death	
17.043	65	regulation of biological quality	
16.853	66	positive regulation of nitrogen compound metabolic process	
16.745	67	regulation of protein modification process	
16.387	70	regulation of intracellular protein kinase cascade	
16.177	72	positive regulation of nucleobase, nucleoside, nucleotide and nucleic acid metabolic process	
16.098	73	positive regulation of intracellular protein kinase cascade	
16.055	74	regulation of biosynthetic process	
15.420	76	positive regulation of cell differentiation	
15.114	77	regulation of cellular protein metabolic process	
14.861	79	regulation of immune system process	
14.694	80	regulation of response to stimulus	
14.693	81	regulation of cellular biosynthetic process	
14.693	82	regulation of MAPKKK cascade	
14.025	84	positive regulation of multicellular organismal process	
13.858	85	regulation of cellular localization	
13.679	87	regulation of protein metabolic process	
13.655	89	regulation of localization	
13.603	90	positive regulation of MAPKKK cascade	
13.471	92	positive regulation of immune system process	
13.247	95	regulation of macromolecule biosynthetic process	
12.864	98	regulation of nucleobase, nucleoside, nucleotide and nucleic acid metabolic process	
12.858	99	positive regulation of transcription from RNA polymerase II promoter	
12.825	100	positive regulation of RNA metabolic process	
42.566	3	anatomical structure development	**Development**
40.853	4	system development	
39.340	7	developmental process	
38.350	10	multicellular organismal development	
37.601	11	organ development	
30.593	14	anatomical structure morphogenesis	
30.178	15	cellular developmental process	
29.715	17	cell differentiation	
18.873	45	nervous system development	

18.180	50	organ morphogenesis	
17.121	62	tissue development	
16.367	71	cell development	
14.999	78	central nervous system development	
14.532	83	cellular component organization	
13.665	88	neurogenesis	
13.528	91	brain development	
13.297	94	generation of neurons	
13.232	96	gland development	
27.193	22	signaling	**Signaling**
19.889	42	signaling pathway	
17.105	63	enzyme linked receptor protein signaling pathway	
16.696	68	signal transmission	
16.696	69	signaling process	
15.820	75	cell surface receptor linked signaling pathway	
13.733	86	signal transduction	
13.455	93	transmembrane receptor protein tyrosine kinase signaling pathway	
38.634	8	response to stimulus	**Stimulus/Stress Response**
22.607	33	response to chemical stimulus	
19.602	43	response to wounding	
17.860	56	defense response	
17.782	58	response to stress	
34.680	13	immune system process	**Immunity/ Inflammation**
29.435	18	immune response	
19.097	44	inflammatory response	
12.916	97	cell activation	
30.040	16	multicellular organismal process	**Cellular**
25.381	27	cellular process	
17.149	61	locomotion	**Motility**
21.874	39	behavior	**Behavior**

SUPPORTING INFORMATION REFERENCES

1. Newman MEJ. The structure and function of complex networks. *SIAM Rev.* 2003;45:167-256.
2. Barabási AL, Oltvai ZN. Network biology: understanding the cell's functional organization. *Nat Rev Genet.* 2004;5:101-113.
3. Shannon P, Markiel A, Ozier O et al. Cytoscape: a software environment for integrated models of biomolecular interaction networks. *Genome Res.* 2003;13:2498-2504.
4. Doncheva NT, Assenov Y, Domingues FS et al. Topological analysis and interactive visualization of biological networks and protein structures. *Nat Protoc.* 2012;7:670-685.
5. Maere S, Heymans K, Kuiper M. BiNGO: a Cytoscape plugin to assess overrepresentation of Gene Ontology categories in biological networks. *Bioinformatics.* 2005;21:3448-3449.
